# Space, time, and dynamics of binocular interactions

**DOI:** 10.1038/s41598-023-48380-2

**Published:** 2023-12-05

**Authors:** Marzouk Yassin, Maria Lev, Uri Polat

**Affiliations:** School of Optometry and Vision Sciences, Faculty of Life Sciences, Bar-Ilan University, Ramat-Gan, Israel

**Keywords:** Neuroscience, Sensory processing, Visual system

## Abstract

Binocular summation (BS), defined as the superiority of binocular over monocular visual performance, shows that thresholds are about 40% (a factor of 1.4) better in binocular than in monocular viewing. However, it was reported that different amounts of BS exist in a range from 1.4 to 2 values because BS is affected by the spatiotemporal parameters of the stimulus. Lateral interactions can be defined as the neuron’s ability to affect the neighboring neurons by either inhibiting or exciting their activity. We investigated the effect of the spatial and temporal domains on binocular interactions and BS under the lateral masking paradigm and how BS would be affected by lateral interactions via a lateral masking experiment. The two temporal alternative forced-choice (2TAFC) method was used. The stimuli consisted of a central vertically oriented Gabor target and high-contrast Gabor flankers positioned in two configurations (orthogonal or collinear) with target-flanker separations of either 2 or 3 wavelengths (λ), presented at 4 different presentation times (40, 80, 120, and 200 ms) using a different order of measurements across the different experiments. Opaque lenses were used to control the monocular and binocular vision. BS is absent at close distances (2λ), depending on the presentation time’s order, for the collinear but not for the orthogonal configuration. However, BS exists at more distant flankers (collinear and orthogonal, 3λ). BS is not uniform (1.4); it depends on the stimulus condition, the presentation times, the order, and the method that was used to control the monocular and binocular vision.

## Introduction

Binocular vision refers to a visual mode that occurs when two eyes are used simultaneously^1^. Normal binocular vision occurs when the brain combines the information it receives from both eyes and requires two normal monocular visual inputs and a normal development of neural connectivity^2,3^. Monocular or binocular abnormalities, such as amblyopia, during the early period of development, lead to deficient binocular vision^4–7^.

In normal vision, binocular summation (BS) is defined as the superiority of binocular over monocular visual performance^8–10^; it indicates that contrast detection and luminance thresholds are about 40–60% better in binocular than in monocular viewing^11–18^. BS decreases with increasing interocular differences in visual acuity^9^. It has been shown that BS is absent in cases of abnormal binocular development such as amblyopia; it decreases as the magnitude of the difference between the eyes increases^6,7,19–21^. Interestingly, it has been found that BS is affected by crowding, tagging^22^, and context^23^.

BS is dependent on contrast. The contrast sensitivity function (CSF) is defined as the ability to detect fine changes in luminance^24^. Studies agree that BS occurs only for small differences between the eyes’ performance^9^. For high contrast (above 15%), monocular and binocular thresholds are approximately equal. BS for low-contrast stimuli is reduced with increasing presentation time. The interocular suppression for low contrast and short presentation times is small; it increases as the contrast increases^25^. However, in orientation discrimination^26^, the interocular suppression decreases as the contrast increases^27^. It was shown that at high spatial frequencies and longer presentation times^28^ the BS is higher^10^.

Early studies of BS found an improvement of a factor of about 1.4, leading to models suggesting a quadratic summation of the two monocular inputs (√2)^10,29–34^. In contrast sensitivity, BS refers to the equation of CSbin = sqrt(CSright^2^ + CSleft^2^)^35^. A recent review^10^ showed that different amounts of BS exist in a range from √2 to 2 values^19^ because the amount of BS is affected by the spatiotemporal parameters of the stimulus. In the last decades, several models of BS have been proposed (see Ref.^1,19,27,32,33,36–45^). Studies suggested a gain control theory^25,32,46^ based on studies by Cogan^33^ and Wilson^47^. They suggested that each eye exerts gain control on the other eye’s signal in proportion to the contrast energy of its own input; moreover, each eye exerts gain control on the other eye’s gain control^25,32,46^. Other studies suggested models with gain enhancement^48,49^, suggesting that the contrast detection facilitation at threshold levels induced by cross-orientation masks with a single free parameter for gain enhancement across all spatiotemporal conditions and eyes. Baker et al.^10^ defined a measure of “stimulus speed” that they calculated as the ratio between stimulus temporal (the presentation time) and the spatial frequency. According to this ratio, a slow speed (including high spatial and low temporal frequencies) will lead to a higher BS^10^.

A recent study^23^, which investigated the BS under stimuli with context, showed no BS of the target during close distances (collinear 2λ) because it suppresses detection more under binocular compared with monocular viewing, but not for the orthogonal configuration. In contrast, more distant collinear configuration (collinear 3λ) facilitates both monocular and binocular detection; hence BS exists. The authors^23^ compared their model with other models; they suggested an updated gain control model. In summary, models^1,19,25,32,33,35,46,47^ of BS have been elaborated for isolated stimuli in the last decades, whereas other models^23,48–62^ of BS have been elaborated for stimuli with context.

The ability of a neuron to affect its neighboring neurons by either exciting or inhibiting their activity is known as lateral interactions^63^. The results of these actions are called facilitation and suppression^63–84^. Physiological observations indicate that they result from a network of long-range connections that exist between similar orientation columns^73–75^. A specific case of lateral interactions, known as collinear facilitation, is characterized by an improved detectability of a Gabor patch by the presence of high-contrast collinear flankers^63–65,81^. The facilitation of target detection increases when flankers are separated from the target by three wavelengths (ƛ), and it decreases for longer distances. Thresholds are elevated (suppression) for shorter target-to-flanker separations. The choice of the target-flanker distance is determined by considering the expected masking effect on the target^63,66,68,85^. The reasons for using 3λ separation support the hypothesis that separations of 3λ or more activate collinear facilitation between different neurons (with no or minimal overlapping between perceptive fields) responding to the target and the mask. It was shown that collinear facilitation involves horizontal connections between cells of similar orientation preference within the primary visual cortex (V1)^76,86^.

It is well known that LM can either facilitate or suppress detection, depending on the distance from the target and the global configuration^63,64,68,87,88^. In binocular vision, at close distances (collinear 2λ), there are two types of suppression: one type induced by the lateral interactions, and the other one induced by interocular suppression. Thus, when the two types of the suppression act together, detection is suppressed more under binocular than under monocular viewing^23,89^. In contrast, more distant flankers (collinear 3λ) facilitate both monocular and binocular detection^23,89^; thus, the interocular suppression is eliminated by the collinear facilitation.

In general, several studies have shown that binocular conditions might not be better than monocular conditions in cases of contrast sensitivity^90–92^, crowding^22,93^, and masking^23,91^. The question of whether two eyes are better than one in cases of contrast sensitivity^23,91,92^, crowding^22^, and masking^23,91^ has been explored in our lab. Moshkovitz et al.^92^ found that two eyes are not always better than one under the spatial–temporal properties of nystagmus perception and that the BS mechanism was impaired in nystagmus subjects. They examined the spatial–temporal aspects of nystagmus perception, aiming to investigate the mechanisms underlying the deterioration of their visual performance under monocular and binocular conditions^92^. Subjects were asked to detect Gabor at different frequencies and presentation times. It was found that the BS was impaired in most of the nystagmus subjects. Importantly, recent studies^23,89,91^ in our lab investigated the perception of binocular vision during contextual modulation (changes in the appearance of target patterns when presented within a surrounding pattern). Lev et al.^23^ investigated the BS phenomenon; it was found that at close distances collinear (but not orthogonal) configuration suppresses detection more under binocular than under monocular viewing. In contrast, more distant collinear configuration facilitates both monocular and binocular detection. Serero et al.^91^ reported that no significant BS of collinear facilitation was observed in cases of both distorted (oblique astigmatism) and non-distorted (normal) vision. The study explored the perception of binocular vision and the target contrast detection of Gabor patches and two collinear flankers at different orientations (180°, 45°, 90°, and 135°) in cases of both distorted (oblique astigmatism) and non-distorted vision. As a result, no significant BS of collinear facilitation was observed. In addition, Benhaim-Sitbon et al.^89^ reported that binocular fusion disorders impair basic visual processing. Importantly it was found that no binocular advantage exists for collinear facilitation with target-flanker separations of 2, 3, 4 and 6λ. Moreover, at close distances (collinear 2λ) suppression was found for the binocular condition in cases of both distorted (subjects with heterophoria) and non-distorted vision (control subjects with normal vision). Siman-Tov et al.^22^ reported that BS under foveal crowding was significantly reduced and was almost absent during a very short presentation time (40 ms). In summary, the BS phenomenon of collinear facilitation is absent during stimuli with context modulation, whereas it existed during isolated stimuli^23,89,91^.

The mechanism underlying BS remains largely under investigation. The mechanisms under the LM paradigm^63,64,66,67^, which quantifies local spatial^64,65,88,94^ and temporal^68,69^ interactions that very often occur in natural vision in which objects appear in context, was not extensively investigated, since it was found^64,65,95^ that collinear interactions at 3λ affect the collinear interactions at 2λ, leading to reduced lateral suppression. Furthermore, it was reported that practice modifies the range of lateral interactions^96^. The two processes (interactions and practice) lead to the dynamic range of lateral interactions. The binocular interactions are affected by the monocular interaction^23,89^; therefore, BS is also affected by the two processes described above. It has been documented that BS is significantly affected by the spatial and temporal frequency of the stimulus^10^; therefore, in our study we will investigate the effect of spatial and temporal domains on binocular interactions and the BS phenomenon. Here we aim to investigate how binocular interactions, hence BS, are affected by collinear and non-collinear interactions under different space and time conditions using the LM paradigm. Furthermore, the current study includes a comprehensive series of experiments that examine the dynamics of binocular combination and how it is affected by the testing order and practice. This information is important to better understand how BS is affected by binocular interactions, by the testing order and by practice. We hypothesized that BS is not uniform: it depends on the stimuli's presentation time order, practice, and target-flanker separations either at 2 or 3λ. In agreement with our hypothesis, we found that the BS of contrast threshold is absent at close distances (2λ), depending on the presentation time’s order, for the collinear but not for the orthogonal configuration. However, the BS of contrast threshold exists at more distant flankers (collinear and orthogonal, 3λ).

## Methods

### Participants

A total of 27 healthy participants were enrolled in the experiments that took place at Bar-Ilan University. The age of the participants was between 18 and 30 years old (27.1 ± 4.98 years, mean ± STD) with normal or corrected-to-normal vision. Each participant was included only after a full optometric eye exam performed by an authorized optometrist that includes visual acuity based on Snellen and log-MAR charts (ETDRS) and refraction (see Table1 in the Supplementary Material for more details). Only participants with healthy eyes, a visual acuity of 6/6 (Log-Mar 0) or better in each eye, and no more than one line difference between eyes, were included and were fully corrected with no amblyopia or ocular disease. Participants with major phoria were excluded. The participants signed a consent form approved by the Internal Review Board (IRB) of Bar-Ilan University, according to the guidelines and regulations for human subject research. All experimental protocols were performed following the guidelines provided by the committee approving the experiments. All participants signed a consent form and received financial compensation for their participation. Participants were recruited using electronic advertisements and direct recruitment.

**Figure 1:**
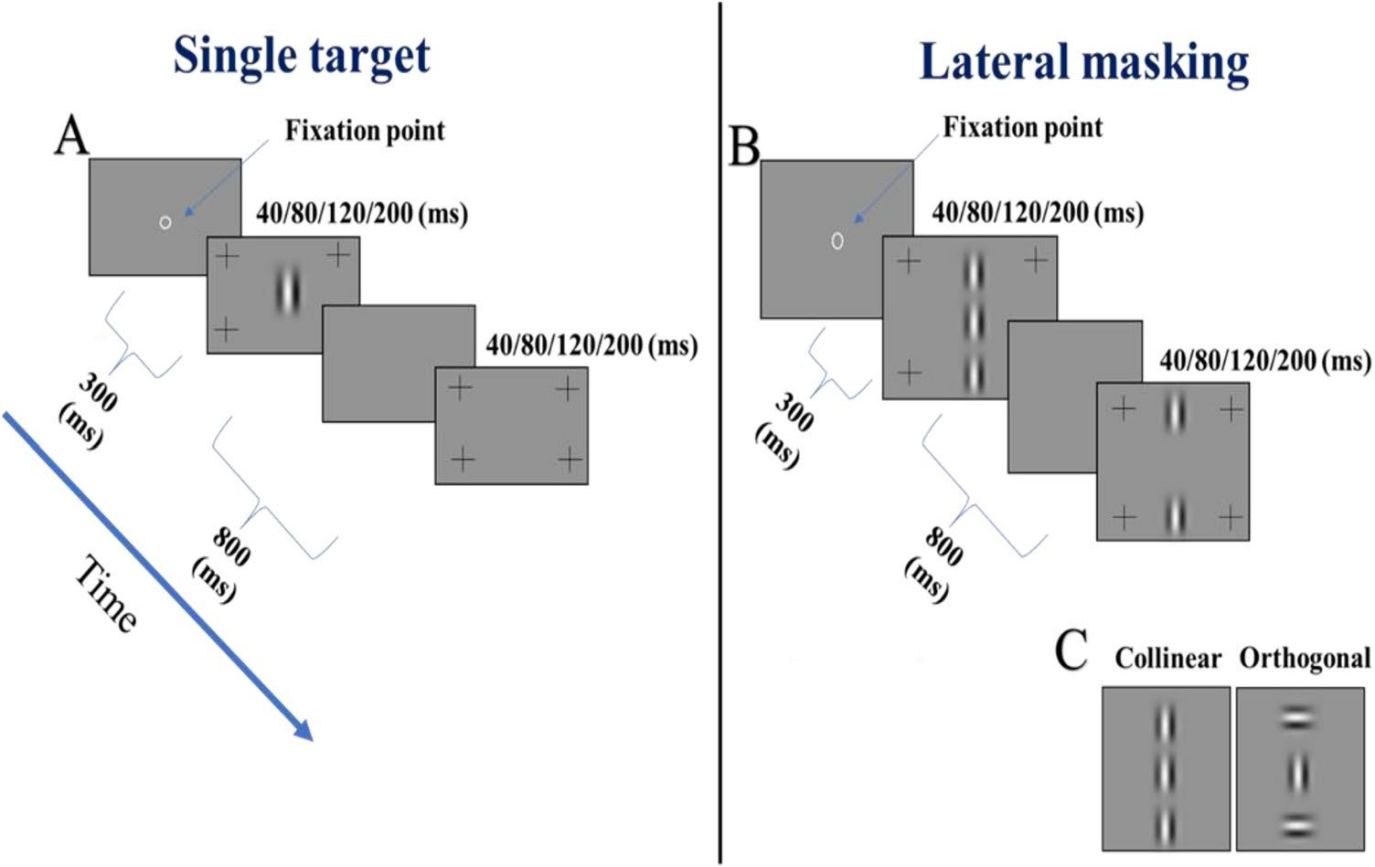
The lateral masking paradigm (LM). Stimuli used in the study. (**A**) Single target: Example of a single Gabor target that was used in the experiments. (**B**) LM paradigm. (**C**) Two configurations that were used in the study Collinear (left) and Orthogonal (right).

### Apparatus

The stimuli were displayed using a PC computer on an EIZO 24 inch FHD with a 100 Hz refresh rate. The screen resolution was 1920 × 1080 pixels, using custom software (PSY, Bonneh, 2004). The effective size of the screen was 52 × 30 cm, which, at a viewing distance of 150 cm, subtended a visual angle of 27.5° and gamma correction was applied.

**Table 1:** Details of the experimental design. MON monocular, BIN binocular, RE right eye, LE left eye, ST single target, COLL collinear configuration, ORTHO orthogonal configuration.

Experiment	Order of the presentation time	Order of the stimuli	Eye’s condition
1	Longer to shorter (200 → 40 ms)	ST → COLL&ORTHO(3λ) → COLL&ORTHO(2λ)	BIN → RE → LE
Control A	Shorter to longer (40 → 200 ms)	ST → COLL&ORTHO(3λ) → COLL&ORTHO(2λ)	BIN → RE → LE
Control B	Longer to shorter (200 → 40 ms)	ST → COLL&ORTHO(2λ) (200 → 40 ms) Finally, COLL&ORTHO(3λ) (200 → 40 ms)	BIN → RE → LE
Control C	Mixed by presentation time (80 → 120 → 40,200 → ms)	Mixed between all the conditions	BIN → RE → LE
Control D	Only 40 ms	ST → COLL(2λ) → COLL(2λ) → COLL(3λ) → COLL(2λ)	MON. before BIN

#### Binocular testing

Experiments were run during either binocular viewing (the two eyes seeing simultaneously) or monocular viewing (one eye was occluded by an opaque lens). Monocular and binocular vision were obtained using right or left opaque lenses with a blocking power of 99.5%, as used in previous studies^89,92,97^. The mean display luminance was 40 cd/m^2^ in an otherwise dark environment.

### The stimuli

The stimuli were presented as gray-level images (Gabor patches, GPs) with an orientation of 90 degrees and with a spatial frequency (SF) of 8 cycles per degree (cpd) with an equal wavelength (λ = 0.21°) and standard deviation (STD, σ); it allows a minimum of 2 cycles in the GP. The stimuli were presented at 4 different presentation times: 40, 80, 120, and 200 ms using different orders of measurements in each experiment. The flanker contrast was 60% or 90%, depending on the presentation time of the stimuli (for a presentation time of 200, 120, and 80 ms, the flanker’s contrast was 60%, whereas for a presentation time of 40 ms the flanker contrast was 90%). The target–flanker orientation differences were either 0°, producing the collinear configuration, or 90°, producing the orthogonal configuration with target–flanker separations of 2 or 3λ. The size of the stimuli for a target-flanker separation of 3λ (center–center) subtends a visual angle of about 1.67° in the central visual field. Each stimulus display included four peripheral high-contrast crosses, marking the interval presentation of the target stimulus (see Fig. 1).

In our study, we decided to use vertical Gabors, as used in our previous studies^23,89,91,92,97^, since we^89,97^ found that subjects with binocular fusion disorders (horizontal phoria) exhibit an abnormal and an asymmetric pattern of both monocular and binocular lateral interactions only for the horizontal meridian, with an absence of collinear facilitation at 3λ, but only for the horizontal meridian. Hence, the phoric subjects exhibited a larger binocular perceptive field size only for the horizontal meridian. In the current study we compared the contrast thresholds of both monocular and binocular viewing; we believe that this approach provides more reliable data. By using the term of 'stimulus condition', we mean that each condition includes subtypes of conditions such as temporal, spatial, or presentation order.

### Procedure

Participants started each trial by pressing the middle mouse button. A visible fixation circle appeared in the center of the screen before each trial and disappeared when the trial started. They were informed of a wrong answer by visual feedback after each presentation throughout the experiment. Each of the experiments consisted of two parts: a single target and a target with flankers (LM paradigm; collinear *vs* orthogonal).The two-temporal-alternative forced-choice paradigm (2TAFC) using a 3:1 staircase procedure known to converge to 79% correct response, were used to measure the target contrast detection threshold^98^. Each trial consisted of two stimuli presented sequentially; only one had a target (the order was randomized). The participants’ task was to determine which stimuli contain the target by pressing the left or right mouse keys (left for the first interval and right for the second).

We measured contrast detection thresholds under monocular and binocular vision for single target (isolated stimuli) or flanked Gabor targets as a function of target–flanker separation of 2 or 3λ (stimuli with context) with spatial frequency of 8cpd (the flanker contrast was 60% or 90%) in either collinear or orthogonal configurations, at 4 different presentation times: 40, 80, 120, and 200 ms with different order of measurement in each experiment (See Table 1 in “Research design and motivations” section for more details). Before the participants started the experiment, there was a practice run of single target matched to the first presentation time which the experiment has been started with.

Each experiment included a total number of 5 blocks (single target, collinear and orthogonal at 2 and 3λ for each configuration), each block consisted of about 40 trials for each condition (about 200 trials per session) for each condition of presentation time. Each data point was repeated 3 times for each eye condition (right, left, binocular), thus the total number of trials for each participant is about 7200 trials. The participants performed the experiments in a total of 12–16 h which divided by an average of 2 h per day for 6–8 different days (depending on the fatigability and the attention capacity of the participant), which the experiment was displayed with different presentation time at each different day.

We calculated the BS as previously calculated in the literature^9,22,23,89,91,92^ as the ratio between the contrast thresholds of the average of two monocular eyes to the binocular threshold. BS Ratio = Monocular/Binocular contrast thresholds for each condition (single target, collinear, orthogonal at 2 and 3λ for each configuration) at 4 different presentation times (40, 80, 120, and 200 ms). We found that there is no significant difference in the contrast threshold between the eyes for each condition during each presentation time during the different experiments, indicating that the performance of both eyes was similar; therefore, "monocular" in this study refers to the mean monocular (see Table 2 in the Supplementary Material for statistical information).

We calculated threshold elevation (facilitation or suppression; collinear and orthogonal) for each eye condition (right, left, binocular) at the 4 different presentation times (at 2 and 3λ) as the log of the ratio between the masked target threshold and the single target threshold, in other words [threshold elevation = log (Masked target threshold/Single Target threshold)] and then we compared the threshold elevation between monocular and binocular conditions for collinear configurations at 2 and 3λ at 4 different presentation times. The specific tasks, conditions and other details are listed in Table 1 (see “Research design and motivations” section).

### Pilot study (mixed by presentation time between the eye’s condition)

We used the ‘mixed’ procedure by blocks of trials to investigate the effect of order on BS at collinear 2λ. Each block consisted of the same five different display conditions: a single target, collinear, or orthogonal configuration displayed at two different separation distances (2 or 3λ). Within each block, the five display conditions were randomized. Furthermore, each block of the five display conditions was presented to the participants at 4 different presentation times, which were randomized across blocks of trials. All 4 presentation times were applied using 3 repetitions to each block of trials. This procedure was randomized across the right, left (monocular), and binocular conditions. Two participants took part in this pilot experiment. Our data of this procedure were inconsistent, very noisy, and do not show a trend or directionality. We thus concluded that in the random order and ‘‘Mixed’’ procedure, with too much randomization, the participants do not apply a constant processing/response strategy. Therefore, when the experiment was presented to participants utilizing the ‘mixed’ method, they were confused, leading to inconsistency of data. In this method the contrast thresholds were different when the measurements were repeated. Thus, we decided to use a constant order of testing during the experiment. The presentation order is detailed in each experiment.

### Research design and motivation

Several variables may affect the binocular interactions and BS (and may not be properly clarified): the spatial condition, presentation time, and testing order. Our hypothesis and the study design are based on several recent studies^22,23,89,91,92^ from our lab showing that BS is affected by the spatial design, i.e., context. One of the emerging hypotheses is that local suppression near the perceptive field may abolish BS. Previous studies^63–65^ showed that collinear interactions at 3λ affect the collinear interactions at 2λ, which consequently affects the contrast threshold. To determine whether BS will exist under the collinear 2λ condition, we tested BS under a few different spatial conditions: collinear (2λ, 3λ) and control for orthogonal (2λ, 3λ). Since we found that BS for 2λ is dynamic, we performed several control experiments to determine how BS may be affected by the other variables, namely, the presentation time and testing order. It was shown that the presentation time affects BS^92^. Thus, we tested the collinear 2λ condition at all presentation times with a gradual order, from the longer to the shorter presentation time and in opposite order for the control. It is also known that practice (short and long training) affects the contrast threshold. Thus, another important control was to determine how ordered and non-ordered presentation times (e.g., mixed and random) affect the BS. This may provide additional insights into the possible effect of decision criteria on BS.

#### Similarities and the differences between the experiments

In each experiment we explored one variable: how changing one parameter (i.e., temporal duration, testing order, and spatial distance) affects the BS while keeping the others constant. To explore the temporal domain of binocular interactions and BS, we tested 4 different presentation times: 40, 80, 120, and 200 ms while the order and sequence of the presentation time were used as variables in each experiment (see Table 1). Another parameter was the spatial configuration (collinear 2λ, 3λ; orthogonal 2λ, 3λ).

### Data and statistical analysis

One-way, Two-way, and Three-way mixed ANOVA were performed to test the effect of 1, 2, or 3 nominal variables (such as presentation time, eye condition, and stimulus condition) on continuous outcomes (contrast threshold, threshold elevation, or BS ratio). Specifically, linear mixed effect models were performed, and the ANOVA was performed on the resulting models. All nominal variables were defined as fixed effects, and the participant’s ID was defined as a random effect. All interactions were included in the initial models; however, if the interactions were non-significant, we refitted the models without these interactions. Post-hoc analysis was performed as pairwise comparisons defined by linear contrasts, and Benjamini-Hochberg (FDR) correction was applied to control for multiple testing. If the interactions were removed, we performed the post-hoc analysis by averaging the non-interacting factors. When the outcome variable was ratios, a logarithm transformation (with base 2) was applied. The normality of residuals and the homogeneity of variance assumptions were assessed graphically with diagnostic plots. All data points were confirmed for not being outliers. All analyses were conducted in the R statistical environment (R Core Team (2021). R: A language and environment for statistical computing. R Foundation for Statistical Computing, Vienna, Austria. URL https://www.R-project.org/.).

## Results

In our study we investigated the effect of spatial and temporal domains on BS using LM paradigm^63^, focusing on monocular and binocular interactions under different space and time conditions.

### Experiment 1: longer to shorter presentation time

**Figure 2:**
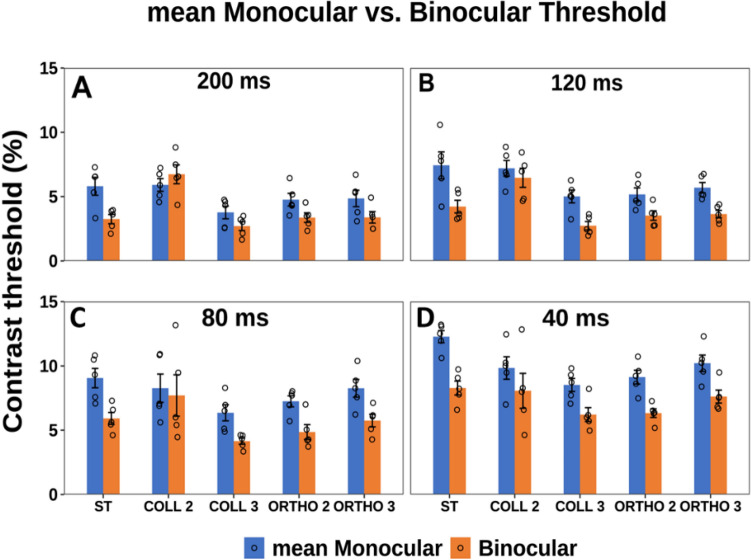
The mean monocular and binocular thresholds according to presentation times of 40, 80, 120, and 200 ms under the LM paradigm. Single Target (ST), Collinear configuration (COLL), Orthogonal configuration (ORTHO) with target–flanker separations of 2 and 3λ for each configuration. (**A**) 200 ms. (**B**) 120 ms. (**C**) 80 ms. (**D**) 40 ms. N = 5, Error bars represent the standard error of the mean (SEM).

First, we measured monocular and binocular contrast thresholds for 4 different presentation times using gradual order, from the longer to the shorter presentation time, for single target (isolated stimuli) and target under LM paradigm (stimuli with context). Figure 2 presents the contrast thresholds of Gabor targets, under monocular and binocular conditions. Importantly, at both single target and collinear 3λ conditions higher contrast thresholds were found for the shorter presentation time whereas a lower contrast threshold was found for the longer presentation time at both monocular and binocular conditions. Please note that we can see a decrease in contrast threshold (improvement) as a function of increasing the presentation time of the target, hence, there is an improvement in contrast thresholds by a factor of 1.4 at both monocular and binocular conditions as a function of the longer presentation time, however it reaches a saturation at 120 and 200 ms. Our results are consistent with the literature^66,99^. Interestingly, this effect is absent at collinear 2λ condition; in other words, there is no improvement in contrast threshold at collinear 2λ condition at both monocular and binocular viewing as a function of the longer presentation time (see Figs. S4–7 in Supplementary Material). In summary, consistent with previous studies^66,92,99^, when investigating how presentation time influences contrast threshold, we found a decrease (improvement) in contrast threshold as a function of the longer presentation time of the target.

#### Monocular versus binocular contrast threshold

We performed a three-way ANOVA to analyze the effect of presentation time, stimulus condition, and group (monocular or binocular viewing) on the contrast detection threshold. There was a significant effect of presentation time on contrast threshold ([F (3,156) = 113.74, *p* = 0.00], stimulus condition [F (4,156) = 28.96, *p* = 0.00] and group (monocular or binocular viewing) [F (1,156) = 128.8, *p* = 0.00]. There was a significant interaction between the effect of presentation time and group (monocular or binocular viewing) [F (3,156) = 3.42, *p* = 0.02], also between condition and group [F (4,156) = 5.84, *p* = 0.0002]. Specifically, for single target condition, the binocular contrast threshold is significantly lower than the monocular at all presentation times 200, 120, 80, 40 ms (*p* = 0.0068, *p* = 0.0007, *p* = 0.0008, *p* = 0.00, by Tukey’s post hoc analysis of 2-way ANOVA, respectively), which could be explained by the BS effect as expected from previous studies^9,100^ (see Fig. 2).

#### Monocular versus binocular lateral interactions

We compared monocular and binocular interactions at collinear configuration for all presentation times at target-flanker separations of 2 and 3λ. We found that there is binocular suppression at collinear 2λ condition which is higher than the monocular suppression for all presentation times. Specifically, there was a significant difference between monocular and binocular viewing at close distances of collinear 2λ condition for presentation times of 200 and 120 ms (*p* = 0.00, *p* = 0.01, by Tukey’s post hoc analysis of 2-way ANOVA, respectively).

A two-way ANOVA was performed to test the effect of presentation time and stimulus condition on threshold elevation. There was a significant effect of presentation time [F (3,60) = 6.47, *p* = 0.0007] and stimulus condition [F (3,60) = 62.28, *p* = 0.00] on threshold elevation. There was a significant interaction between the effect of presentation time and stimulus condition [F (9,60) = 3.35, *p* = 0.002]. Specifically, there was a significant difference between monocular and binocular viewing at close distances of collinear 2λ condition for presentation times of 200 and 120 ms (*p* = 0.00, *p* = 0.01, by Tukey’s post hoc analysis of 2-way ANOVA, respectively). This result suggests that suppression of detection is higher under binocular compared to monocular viewing, probably due to effect of interocular suppression. However, during the shorter presentation times (80 and 40 ms), the suppression at collinear 2λ condition was decreased in both monocular and binocular vision and there is no significant difference between monocular and binocular viewing (*p* = 0.16, *p* = 0.24, by Tukey’s post hoc analysis of 2-way ANOVA, respectively).

#### BS Phenomenon

**Figure 3:**
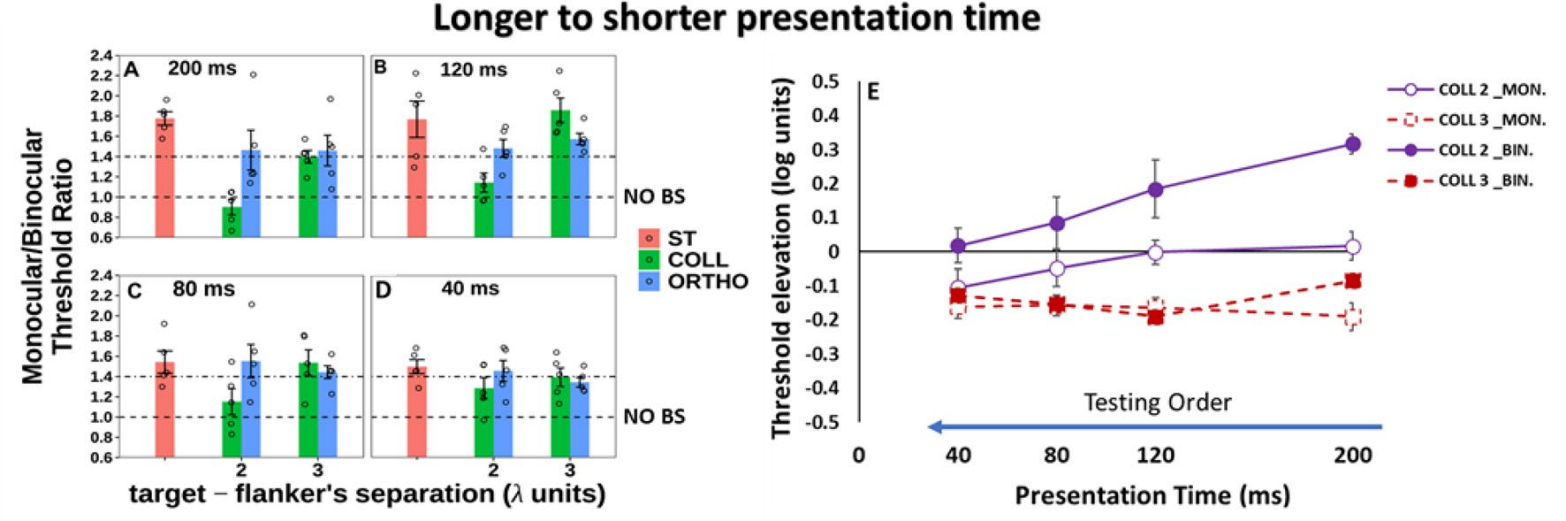
(**A**–**D**) Binocular summation factor (the monocular/binocular threshold ratio) according to presentation times of 40, 80, 120,and 200 ms under the LM paradigm. Single target (ST), Collinear configuration (COLL), Orthogonal configuration (ORTHO) with target–flanker separations of 2 and 3λ for each configuration. (**A**) 200 ms. (**B**). 120 ms. (**C**). 80 ms. (**D**) 40 ms.N = 5, Error bars represent the standard error of the mean (SEM). (**E**) Collinear Interactions as a function of the presentation time (40, 80, 120, and 200 ms). MONOCULAR (MON.), BINOCULAR (BIN.), Collinear configuration (COLL) with target–flanker separations of 2 and 3λ. Facilitation is indicated by values below zero, and suppression by values above zero. N = 5.Error bars represent the standard error of the mean (SEM). The blue arrow points to the left from longer to shorter presentation time describe the testing order.

The results of BS are presented in Fig. 3. We found BS above the ratio = 1, at almost all presentation times for all conditions under collinear and orthogonal of 3λ conditions. However, our results did not show BS effect in collinear 2λ at 200 ms showing that BS < 1. Importantly, for the control condition (orthogonal 2λ) the BS was ≥ 1.4 and almost equal to the single target for all presentation times. Interestingly, we found that binocular viewing confers an advantage over monocular viewing also at close target-flaker distances for the shorter presentation times. This result may be explained because the monocular contrast threshold is higher than the binocular contrast threshold at collinear 2λ in shorter presentation times. Interestingly, please note that only at the longer presentation time of 200 ms, there is no BS for collinear 2λ; however, this could be explained by the effect of the testing order.

We performed a two-way ANOVA to assess the effect of presentation time and stimulus condition on BS ratio. We found a significant effect of stimulus condition on BS ratio [F (4,76) = 15.6, *p* = 0.00]; however, no significant effect of presentation time on BS ratio was found [F (3,76) = 2.46, *p* = 0.07]; nor a significant interaction between presentation time and stimulus condition [F (12,76) = 1.6, *p* = 0.1].

To test the effect of spatial configuration on the BS, we measured the effect with orthogonal configuration at target-flanker separations of 2 and 3λ as a control test across all different experiments. We found that under these conditions the results are similar to those under a single target, showing that BS exists, indicating that there is no significant difference between single target and orthogonal configurations at target-flanker separations of 2 and 3λ (*p* = 0.30, *p* = 0.19, by Tukey’s post hoc analysis of 2-way ANOVA, respectively); Therefore, in the results of the next experiments, for simplicity, we presented only for the results at collinear configuration in the Figures.

The results of collinear 3λ were uniform across different experiments, showing collinear facilitation for both monocular and binocular vision as well as BS similar to a single target, which is consistent with previous studies^23,89^. However, we continue to present this information, since it was found^64,65,95^ that it affects the collinear interactions at 2λ.

In the next experiments, we aimed to investigate how the testing order affects binocular interactions and BS.

### Control experiment A: shorter to longer presentation times

Experiment 1 showed an absence of BS for collinear 2λ at 200 ms, which was the first testing session of presentation time. This effect decreased after the next presentation times; therefore, we performed a control experiment to investigate how the testing order affects binocular interactions and the BS phenomenon. The stimuli were displayed from shorter to longer presentation times: 40, 80, 120, and 200 ms. A total of 5 new participants were enrolled in this control experiment.

#### Monocular versus binocular lateral interactions

**Figure 4:**
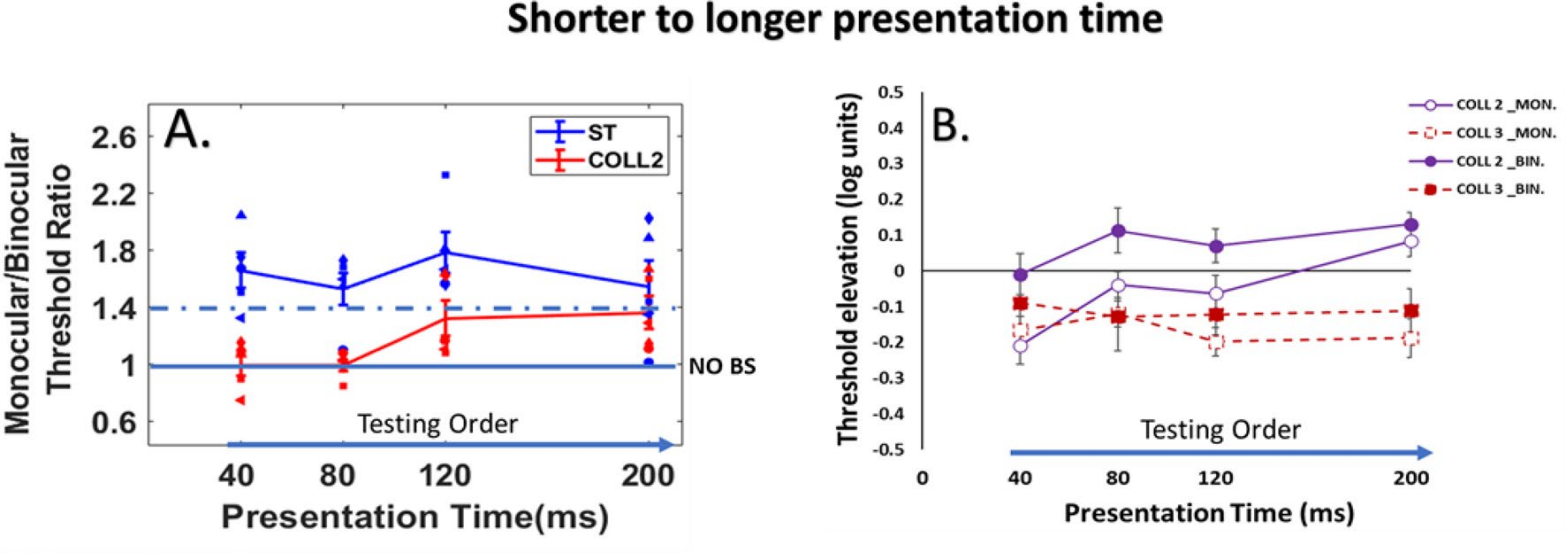
(**A**) Binocular summation factor (the monocular/binocular threshold ratio) according to presentation times of 40, 80, 120, and 200 ms under the LM paradigm. Single target (ST), Collinear configuration with target–flanker separations of 2λ (COLL2). N = 5, Error bars represent the standard error of the mean (SEM). (**B**) Collinear Interactions as a function of the presentation time (40, 80, 120, and 200 ms). MONOCULAR (MON.), BINOCULAR (BIN.), Collinear configuration (COLL) with target–flanker separations of 2 and 3λ. Facilitation is indicated by values below zero, and suppression by values above zero. N = 5.Error bars represent the standard error of the mean (SEM). The blue arrow points to the right from shorter to longer presentation time describe the testing order.

For binocular viewing, detection is facilitated by collinear flankers at a separation of 3*λ* and strongly suppressed by collinear flankers at close distances 2*λ* (see Fig. 4B), consistent with previous studies^23,63,89^. Interestingly, for monocular viewing, there is similar collinear facilitation at 3*λ* as the binocular condition, however, at close distances collinear 2*λ,* binocular suppression is significantly higher than the monocular suppression (*p* = 0.0006, by Tukey’s post hoc analysis of 2-way ANOVA). There was a significant effect of presentation time [F (3,60) = 3.50 *p* = 0.02, two-way ANOVA] and stimulus condition [F (3,60) = 21.29, *p* = 0.00, two-way ANOVA] on threshold elevation. There was no significant interaction between the effect of presentation time and stimulus condition [F (9,60) = 2.03, *p* = 0.0503, two-way ANOVA]. Specifically, there was a significant difference between monocular and binocular viewing at collinear 2*λ* condition (*p* = 0.0006, by Tukey’s post hoc analysis of 2-way ANOVA). Please note that our results showing increasing of the suppression at collinear configuration in both monocular and binocular vision for close distances (collinear 2λ) at longer presentation times.

#### BS phenomenon

Here, we found the opposite effect compared to the results of experiment 1. The BS results are presented in Fig. 4A. We found, BS for all conditions of the single target, collinear 3λ, and orthogonal 2 and 3λ; However, at shorter presentation times of 40 and 80 ms, BS is absent for collinear 2λ condition whereas it exist at the longer presentation times of 120 and 200 ms. Importantly, our results show that the order of presentation time affects the binocular interactions and BS; no BS effect was found for collinear 2λ condition at shorter presentation times 40 and 80 ms, probably because it was the first conditions of testing. In other words, in this presentation order, we found that binocular viewing confers an advantage over monocular viewing also at close distances for the longer presentation times 120 and 200 ms. We found a significant effect of stimulus condition on BS ratio [F (4,76) = 9.48, *p* = 0.00, two-way ANOVA]; however, no significant effect of presentation time on BS ratio was found [F (3,76) = 1.21, *p* = 0.31, two-way ANOVA]; nor a significant interaction between presentation time and stimulus condition [F (12,76) = 1.66, *p* = 0.09, two-way ANOVA].

In summary, we found that the testing order affects binocular interactions and BS at collinear 2λ condition; either the stimuli's presentation time order during the experiment was displayed from the longer to shorter presentation time or vice versa, indeed at collinear 2λ condition BS is not uniform and it depends on testing order, in other words BS is dynamic at collinear 2λ condition, whereas for single target, collinear 3λ, and orthogonal 2 and 3λ conditions BS is uniform.

### Control experiment B: 2λ before 3λ (longer to shorter presentation time)

It was found^64,65,95^ that collinear interactions at 3λ affect the collinear interactions at 2λ, which consequently affect the contrast threshold. In addition, in our experiments (exp.1 and control A) we found that the suppression decreased in collinear 2λ for presentation time of 40 ms, in both monocular and binocular viewing. In both experiments, the stimuli were presented in a random order of target-flanker separations of 2 and 3λ, leading to decreased suppression at collinear 2λ at presentation time of 40 ms, probably due to the order effect. Therefore, we aimed to investigate how the testing order affects binocular interactions and the BS. To determine whether BS will exist only at collinear 2λ condition, we performed a control experiment in which the participants were tested first only in the condition of collinear 2λ, binocularly and monocularly, at all presentation times with a gradual order from the longer to the shorter presentation time. After testing the collinear 2λ condition, participants performed the condition of collinear 3λ. A total of 5 new participants were enrolled in this study.

First, we measured the contrast thresholds of single targets according to 4 different presentation times (see Fig. S2 in Supplementary Material) under monocular and binocular viewing for a spatial frequency of 8cpd.

#### Monocular versus binocular lateral interactions

**Figure 5:**
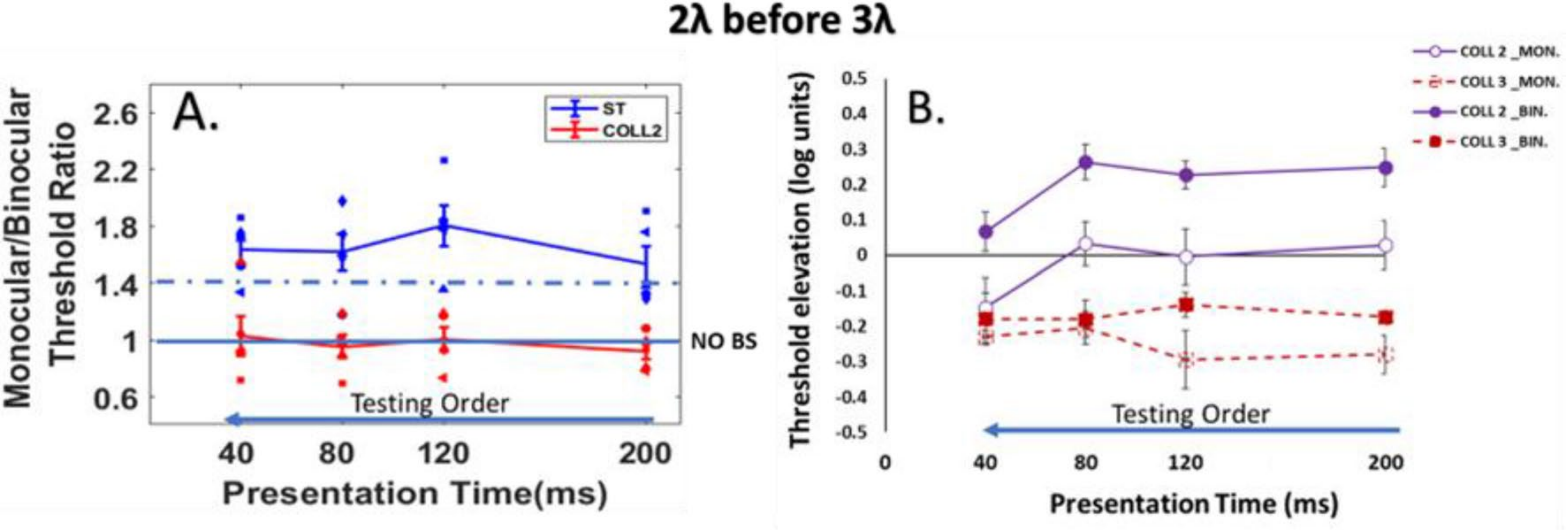
(**A**) Binocular summation factor (the monocular/binocular threshold ratio) according to presentation times of 40, 80, 120, and 200 ms under the LM paradigm. Single target (ST), Collinear configuration with target–flanker separations of 2λ (COLL2). N = 5, Error bars represent the standard error of the mean (SEM). (**B**) Collinear Interactions as a function of the presentation time (40, 80, 120, and 200 ms). MONOCULAR (MON.), BINOCULAR (BIN.), Collinear configuration (COLL) with target–flanker separations of 2 and 3λ. Facilitation is indicated by values below zero, and suppression by values above zero. N = 5.Error bars represent the standard error of the mean (SEM). The blue arrow points to the left from longer to shorter presentation time describe the testing order.

For binocular viewing, detection is facilitated by collinear flankers at a separation of 3*λ* and strongly suppressed by collinear (but not orthogonal) flankers at close distances 2*λ* (see Fig. 5B), consistent with previous studies^23,63,89^. Interestingly, for monocular viewing, there is similar collinear facilitation at 3*λ* as the binocular condition, however at collinear 2*λ,* binocular suppression is significantly higher than the monocular suppression at all presentation times of 200, 120, 80, 40 ms (*p* = 0.03, *p* = 0.02, *p* = 0.02, *p* = 0.03, by Tukey’s post hoc analysis of 2-way ANOVA, respectively). Please note that at collinear 2λ condition only at 40 ms, the suppression decreased in both monocular and binocular viewing, this result is probably due to the order effect.

A two-way ANOVA was performed to test the effect of presentation time and stimulus condition on threshold elevation. There was a significant effect of presentation time [F (3,60) = 3.38, *p* = 0.02] and stimulus condition [F (3,60) = 71.29, *p* = 0.00] on threshold elevation. There wasn’t a significant interaction between the effect of presentation time and stimulus condition [F (9,60) = 1.52, *p* = 0.16]. Specifically, there was a significant difference between monocular and binocular viewing at collinear 2λ condition at all presentation times of 200, 120, 80, 40 ms (*p* = 0.03, *p* = 0.02, *p* = 0.02, *p* = 0.03, by Tukey’s post hoc analysis of 2-way ANOVA, respectively).

#### BS Phenomenon

The results of BS ratio are presented in Fig. 5A. We found BS for the single target, collinear 3λ, and orthogonal 2 and 3λ conditions at all presentation times. However, no BS was found for collinear 2λ condition at all presentation times; here we found suppression at collinear 2λ condition for all presentation times but decreased only at 40 ms in both monocular and binocular viewing. Interestingly, when we performed the collinear 2λ condition before collinear 3λ, there is no BS for collinear 2λ, probably because the order affects the binocular interactions at collinear 2λ condition, which affect BS. We found a significant effect of stimulus condition on BS ratio [F (4,76) = 18.32, *p* = 0.00, two-way ANOVA]; however, no significant effect of presentation time on BS ratio was found [F (3,76) = 0.42, *p* = 0.73, two-way ANOVA]; nor a significant interaction between presentation time and stimulus condition [F (12,76) = 0.92, *p* = 0.52, two-way ANOVA].

### Control experiment C: mixed procedure by presentation time

Here we aimed to investigate how the testing order affects binocular interactions and the BS. To eliminate the effect of repetitions on practice, the experiment was performed using the “Mix” procedure. In the “Mix” procedure, the trials with different target–flanker configurations were presented in a random order. Also, the stimuli were displayed with a random order by presentation time (80, 120, 40, 200 ms). A total of 5 new participants were enrolled in this study.

#### Monocular versus binocular lateral interactions

**Figure 6:**
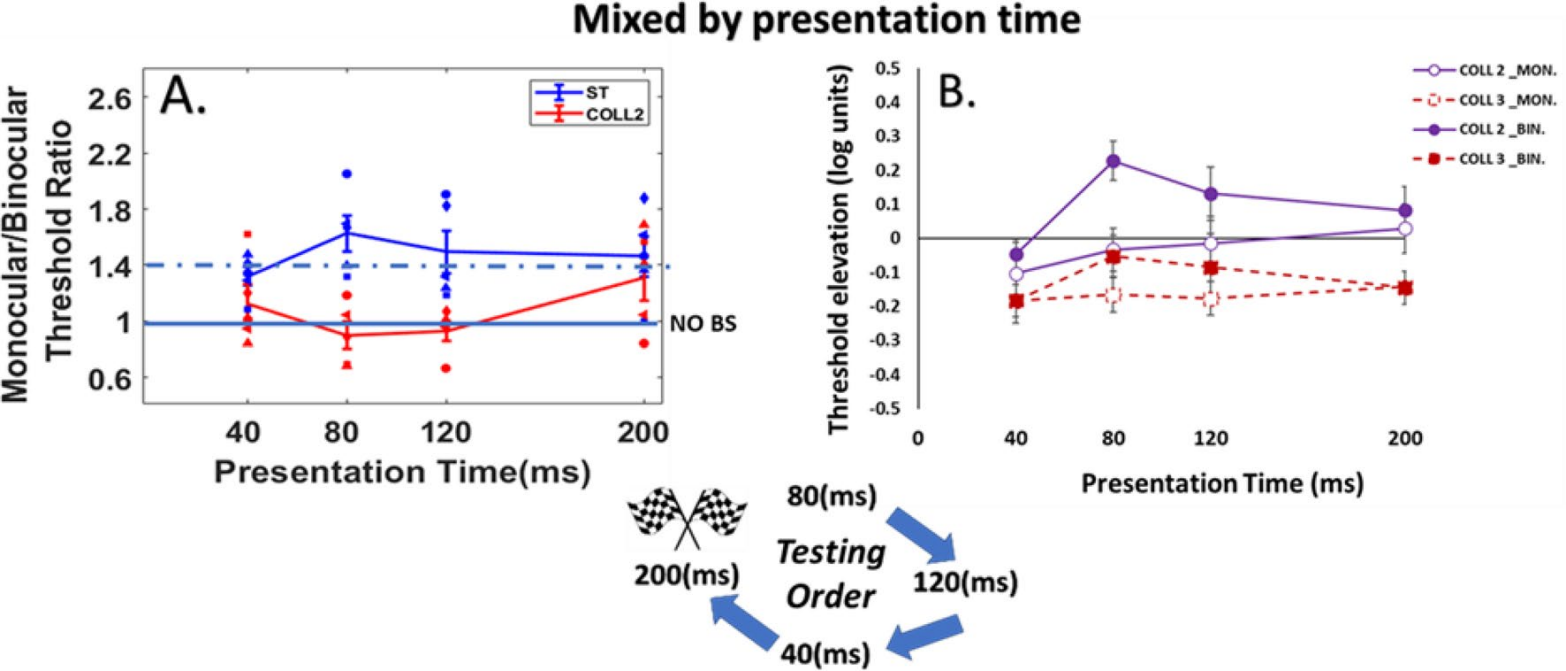
(**A**) Binocular summation factor (the monocular/binocular threshold ratio) according to presentation times of 40, 80, 120, and 200 ms under the LM paradigm. Single target (ST), Collinear configuration with target–flanker separations of 2λ (COLL2). N = 5, Error bars represent the standard error of the mean (SEM). (**B**) Collinear Interactions as a function of the presentation time (40, 80,120, and 200 ms). MONOCULAR (MON.), BINOCULAR (BIN.), Collinear configuration (COLL) with target–flanker separations of 2 and 3λ. Facilitation is indicated by values below zero, and suppression by values above zero. N = 5.Error bars represent the standard error of the mean (SEM). The blue arrows describe the testing order (started at 80 ms and finished at 200 ms).

We found that at close distances of collinear 2*λ,* binocular suppression is significantly higher than the monocular suppression at the first session of 80 ms, however no significant difference for the other presentation times of 120, 40, 200 ms (*p* = 0.002, *p* = 0.25, *p* = 1, *p* = 1, by Tukey’s post hoc analysis of 2-way ANOVA, respectively). Please note that our results showing reduction of the suppression at collinear configuration with close distances (collinear 2λ) for the binocular viewing at presentation times 40, 120, 200 ms (see Fig. 6B); For the monocular viewing the suppression decreased only at 40 ms of presentation time, whereas at the first session of 80 ms we found a strong suppression in both monocular and binocular viewing.

There was a significant effect of presentation time [F (3,60) = 6.52, *p* = 0.0007, two-way ANOVA] and stimulus condition [F (3,60) = 31.60, *p* = 0.00, two-way ANOVA] on threshold elevation. There wasn’t a significant interaction between the effect of presentation time and stimulus condition [F (9,60) = 1.70, *p* = 0.11, two-way ANOVA]. Specifically, there was a significant difference between monocular and binocular viewing at collinear 2λ condition for the first presentation time that was displayed (80 ms) however no significant difference for the other presentation times of 120,40,200 ms (*p* = 0.002, *p* = 0.25, *p* = 1, *p* = 1, by Tukey’s post hoc analysis of 2-way ANOVA, respectively).

#### BS phenomenon

The results of BS are presented in Fig. 6A. Interestingly, only at the first presentation times of 80 and 120 ms, there is no BS for collinear 2λ showing that BS < 1 probably because it was the first presentation times of the stimuli. Interestingly, we found that binocular viewing confers an advantage over monocular viewing also at close distances for the last presentation times (40 and 200 ms) that were displayed, resulting in average of BS ratio between 1.2 and 1.4. This result of BS is probably due to the order effect. Importantly, for the control conditions (orthogonal 2 and 3λ) the BS ratio was ≥ 1.4 and almost equal to the single target for all presentation times. We also found BS effect at all presentation times under collinear 3λ condition.

We found a significant effect of stimulus condition on BS ratio [F (4,76) = 9.34, *p* = 0.00, two-way ANOVA]; however, no significant effect of presentation time on BS ratio was found [F (3,76) = 1.52, *p* = 0.22, two-way ANOVA]; nor a significant interaction between presentation time and stimulus condition [F (12,76) = 1.03, *p* = 0.43, two-way ANOVA].

In summary, we found that the testing order affects binocular interactions and BS at collinear 2λ condition; either the stimuli's presentation time order during the experiment was displayed from the longer to shorter presentation time or vice versa, or mixed by presentation time, neither the order of the stimuli’s conditions were displayed (collinear 2λ before 3λ). Indeed, at collinear 2λ condition, BS is not uniform, and it depends on testing order, in other words BS is dynamic at collinear 2λ condition, whereas at single target, collinear 3λ, and orthogonal 2 and 3λ conditions BS is uniform.

### Control experiment D: monocular before binocular at 40 ms

In our previous experiments (exp.1, control A–C) we found that under the collinear 2λ condition of a presentation time of 40 ms, the suppression decreased in both monocular and binocular viewing probably due to the order effect. Here we investigated how the testing order affects the binocular interactions and the BS. We performed a control experiment with the presentation time of the stimuli only at 40 ms without mixed presentation times or mixed between target-flanker separations of 2 and 3λ (see the order testing below).

**Figure 7:**
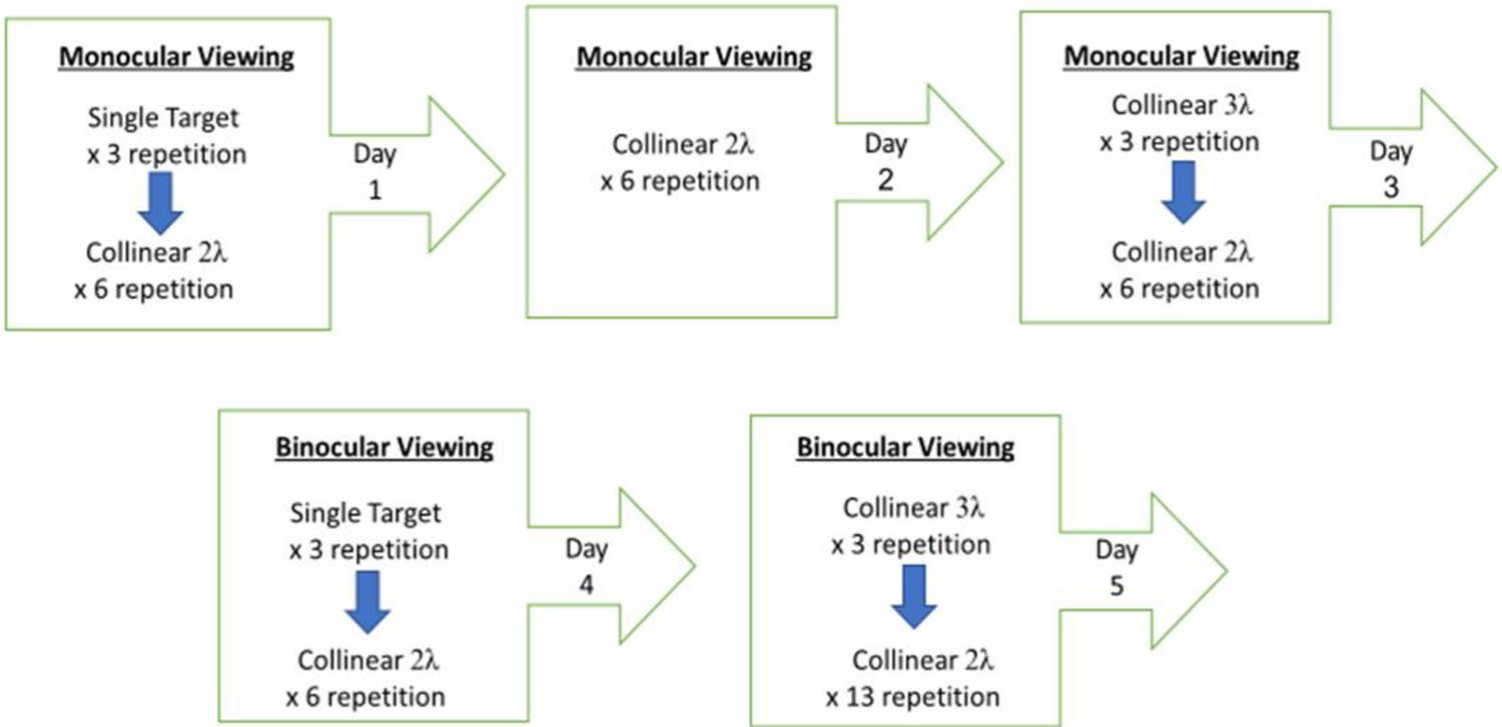
Illustration of the testing order for control experiment D.

**Day 1:** The participants were tested monocularly only in the condition of collinear 2λ before testing 3λ, only at presentation time of 40 ms, to assess if there will be monocular collinear suppression only at collinear 2λ. **Day 2:** They performed again monocularly the condition of collinear 2λ to test if the monocular collinear suppression will decrease at collinear 2λ. **Day 3:** They performed monocularly the condition of collinear 3λ and finally, at the same meeting they performed monocularly the condition of collinear 2λ again, to test the order effect. **Day 4:** only after the participants finished all the conditions and all the runs at the monocular viewing they participated at the binocular viewing; they performed the condition of collinear 2λ before 3λ binocularly to assess if there will be binocular collinear suppression only at collinear 2λ. **Day 5:** In the final meeting they performed binocularly the condition of collinear 3λ and finally at the same meeting they performed binocularly the condition of collinear 2λ to test if the binocular collinear suppression will decrease at collinear 2λ (see illustration in Fig. 7). A total of 5 new participants were enrolled in this study.

#### Monocular versus binocular contrast threshold

**Figure 8:**
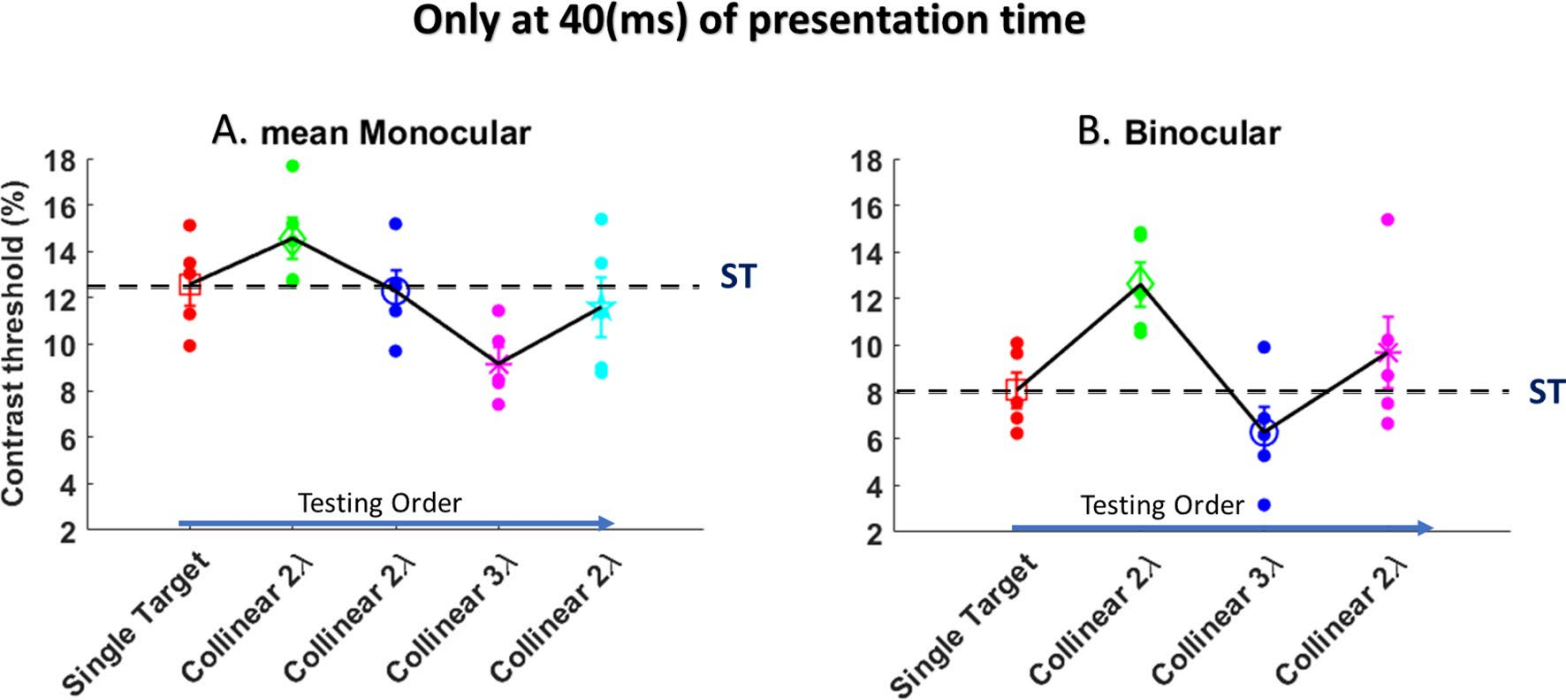
Contrast threshold as a function of the stimulus condition under the LM paradigm. (**A**) The mean Monocular. (**B**) Binocular. N = 5, Error bars represent the standard error of the mean (SEM). The blue arrow points to the right describe the testing order.

The results of contrast threshold for both monocular and binocular viewing at a presentation time of 40 ms are presented in Fig. 8. There was a significant effect of stimulus condition [F (5,48) = 6.8, *p* = 0.000007, two-way ANOVA] and eye condition [F (1,48) = 13.37, *p* = 0.0006, two-way ANOVA] on contrast threshold. There was no significant interaction between the effect of stimulus condition and eye condition [F (5,48) = 0.31, *p* = 0.90, two-way ANOVA], for more statistical information see Table 3 in Supplementary Material.

#### Monocular versus binocular lateral interactions

**Figure 9:**
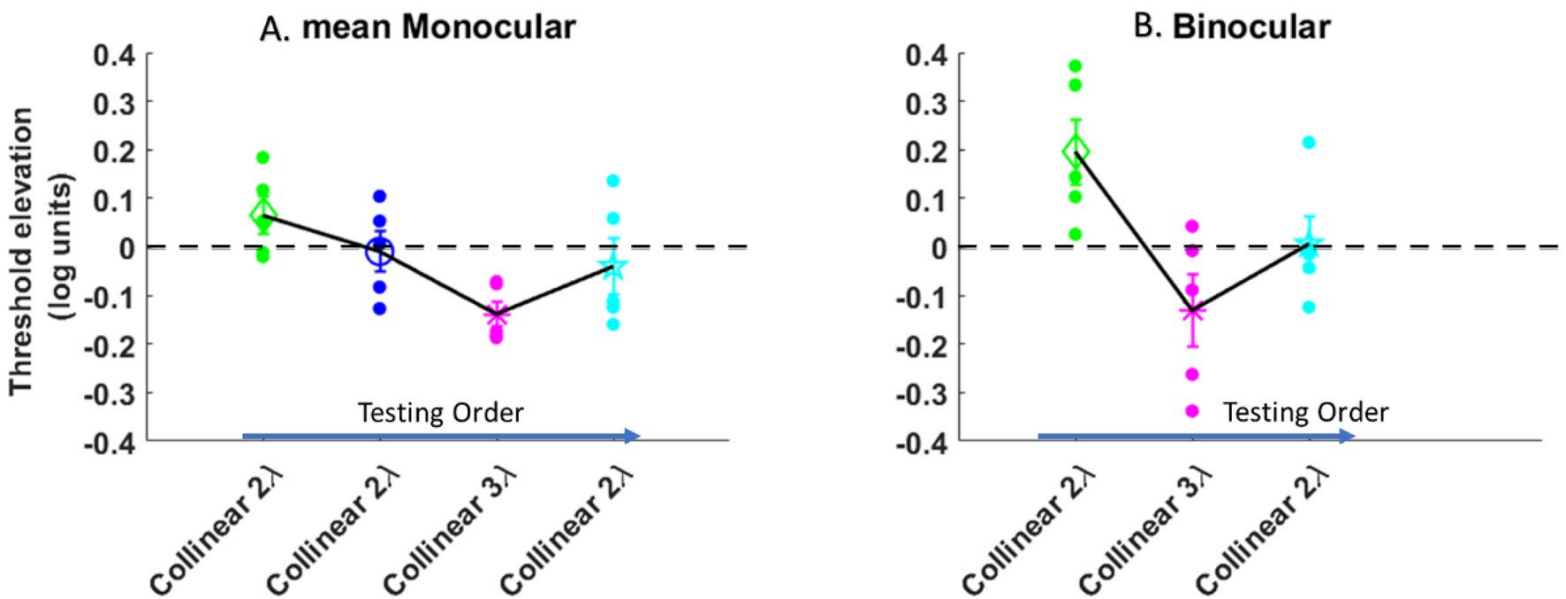
Collinear Interactions at target-flanker separations of 2 and 3λ. (**A**) The mean Monocular. (**B**) Binocular. Facilitation is indicated by values below zero, and suppression by values above zero. N = 5, Error bars represent the standard error of the mean (SEM). The blue arrow points to the right describe the testing order.

The results of collinear interactions for both monocular and binocular viewing at a presentation time of 40 ms are presented in Fig. 9.

We found collinear suppression at collinear 2λ condition depending on testing order whereas collinear facilitation was found for collinear 3λ condition at both monocular and binocular viewing. Please note that the collinear suppression at collinear 2λ condition decreased at both monocular and binocular viewing depending on testing order, i.e., from the second condition testing (see Fig. 9).

There was a significant effect of stimulus condition [F (2,24) = 11.36, *p* = 0.0003, two-way ANOVA] on threshold elevation, however, no significant effect of eye condition [F (1,24) = 1.84, *p* = 0.18, two-way ANOVA] on threshold elevation. There was no significant interaction between the effect of stimulus condition and eye condition [F (2,24) = 0.64, *p* = 0.53, two-way ANOVA]. Specifically, there was a significant difference between collinear 2λ and 3λ condition (*p* = 0.0008), between collinear 2λ and 2λ condition (*p* = 0.0003), between collinear 3λ and 2λ condition (*p* = 0.01) respectively to the testing order.

#### BS phenomenon

**Figure 10:**
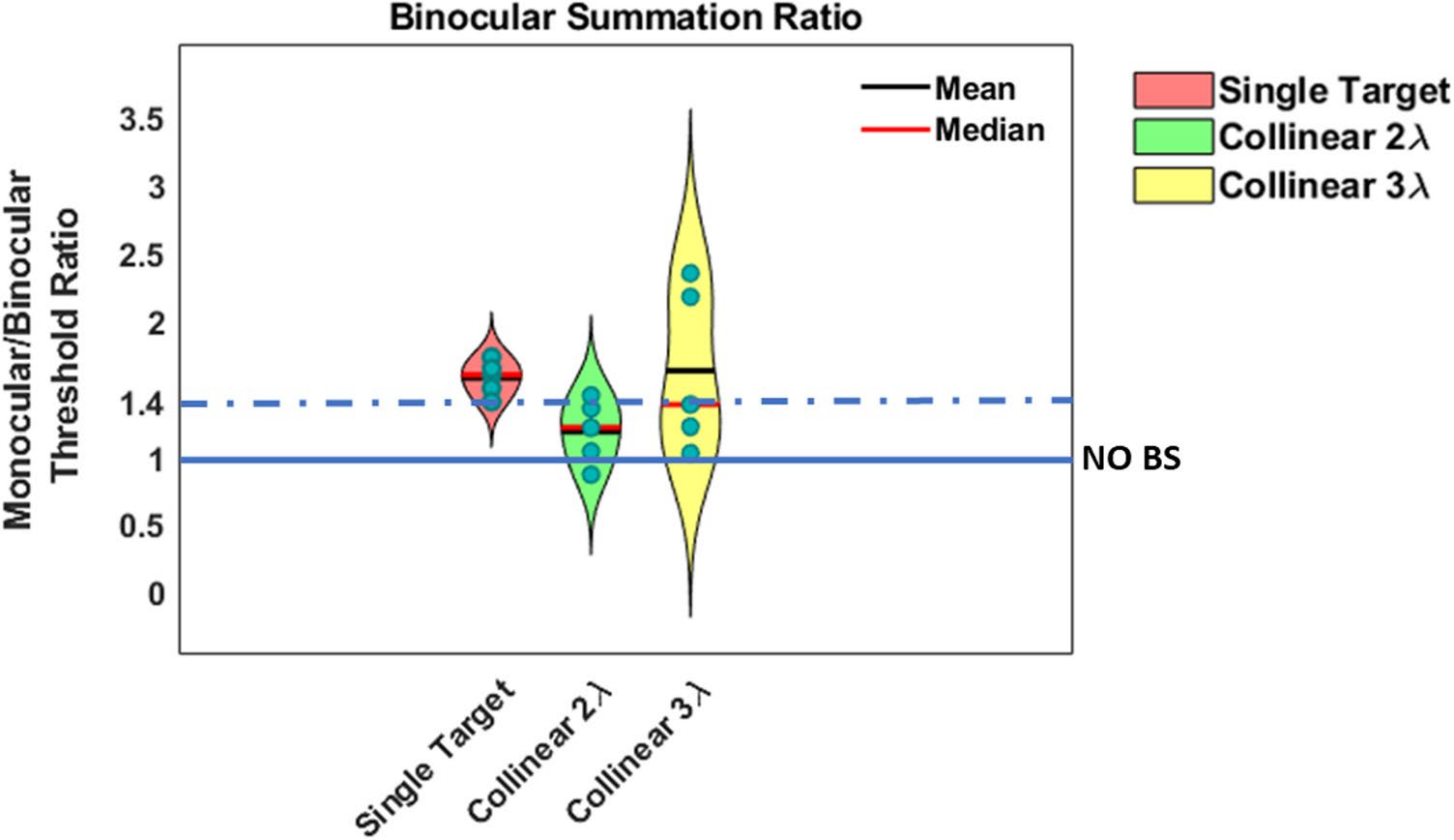
The violin plot shows the ratio of monocular to binocular contrast threshold for isolated stimuli versus stimuli with context, N = 5.

The results of BS are presented in Fig. 10. We found BS at single target and collinear 3λ (BS = 1.57, 1.62, respectively), whereas BS absent at collinear 2λ (BS = 1.18). There was no significant effect of stimulus condition on BS ratio, for more statistical information, see Table 4 in the Supplementary Material.

### Summary across the different experiments (exp.1, control A-C)

**Figure11:**
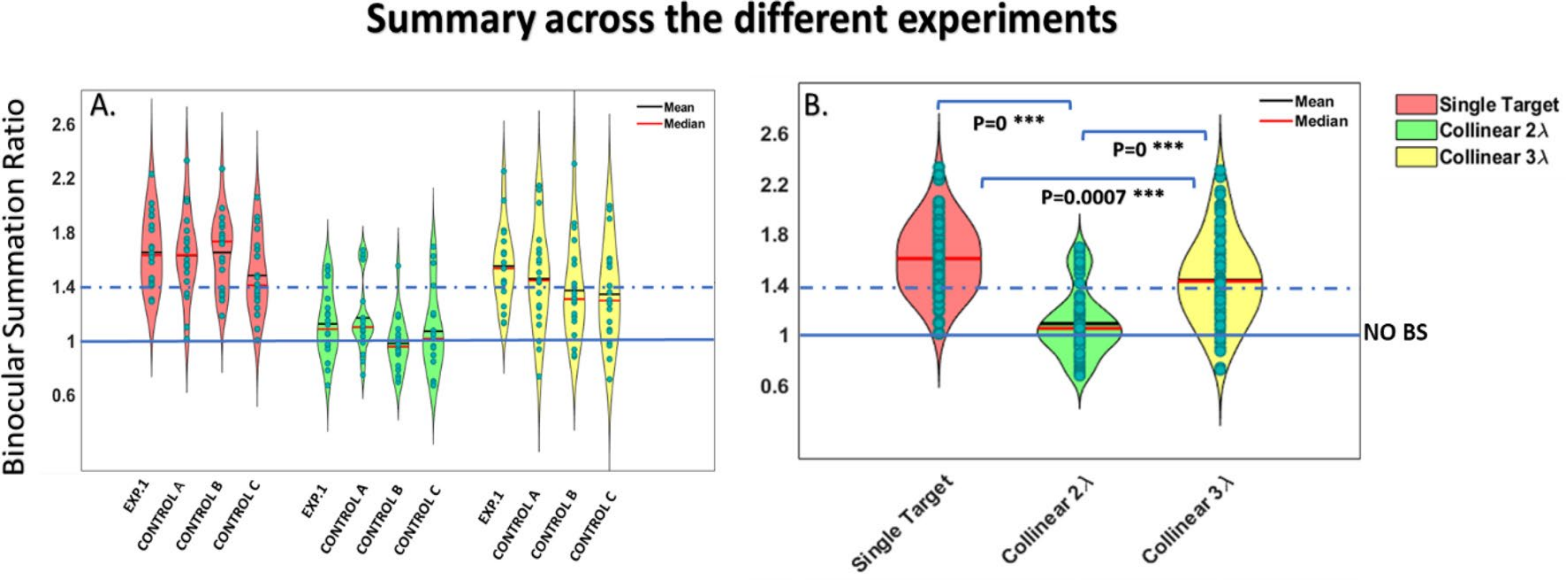
(**A**) The violin plot shows the ratio of monocular to binocular contrast threshold for 20 different participants across the different experiments: experiment1, control A, control B, control C for isolated stimuli vs. stimuli with context at 4 different presentation times (N = 5*4 = 20) (**B**) The violin plot shows the ratio of monocular to binocular contrast threshold for 20 different participants averaged from experiment1, control A, control B, and control C experiments for isolated stimuli vs. stimuli with context at 4 different presentation times (N = 5*4 = 20). We found that the binocular advantage is significantly greater for isolated (Single Target) than for closely flanked stimuli, collinear 2λ (*p* = 0.00***, by Tukey’s post-hoc analysis of 2-way ANOVA).

Figure 11A presents the BS effect as a function of the 4 different experiments for each stimulus condition for all the 4 different presentation times: Since there is no significant effect of presentation time on BS at each individual experiment (*p* = 0.07, *p* = 0.31, *p* = 0.73, *p* = 0.22, two-way ANOVA), we averaged all conditions as a mean value of BS ratio for all the different presentation times for each stimulus condition at each individual experiment (see Fig. 11A).

Figure 11B presents the BS effect as a function of stimulus condition after averaging all the 4 different experiments: Next, we performed a two-way ANOVA to assess the effect of experiment and stimulus condition on BS. Since there is no significant difference of BS between the 4 different experiments for each stimulus condition [F (3,29.97) = 1.63, *p* = 0.20, two-way ANOVA], therefore, these conditions represented the mean value of BS for all the 4 different experiments at each stimulus condition (see Fig. 11B). No significant effect of experiment on BS was found [F (3,29.97) = 1.63, *p* = 0.20, two-way ANOVA]; however; we found a significant effect of stimulus condition on BS [F (2,213.35) = 64.35, *p* = 0.00, two-way ANOVA], nor significant interaction was found between stimulus condition and experiment [F (6,213.35) = 0.94, *p* = 0.46, two-way ANOVA].

**Figure 12:**
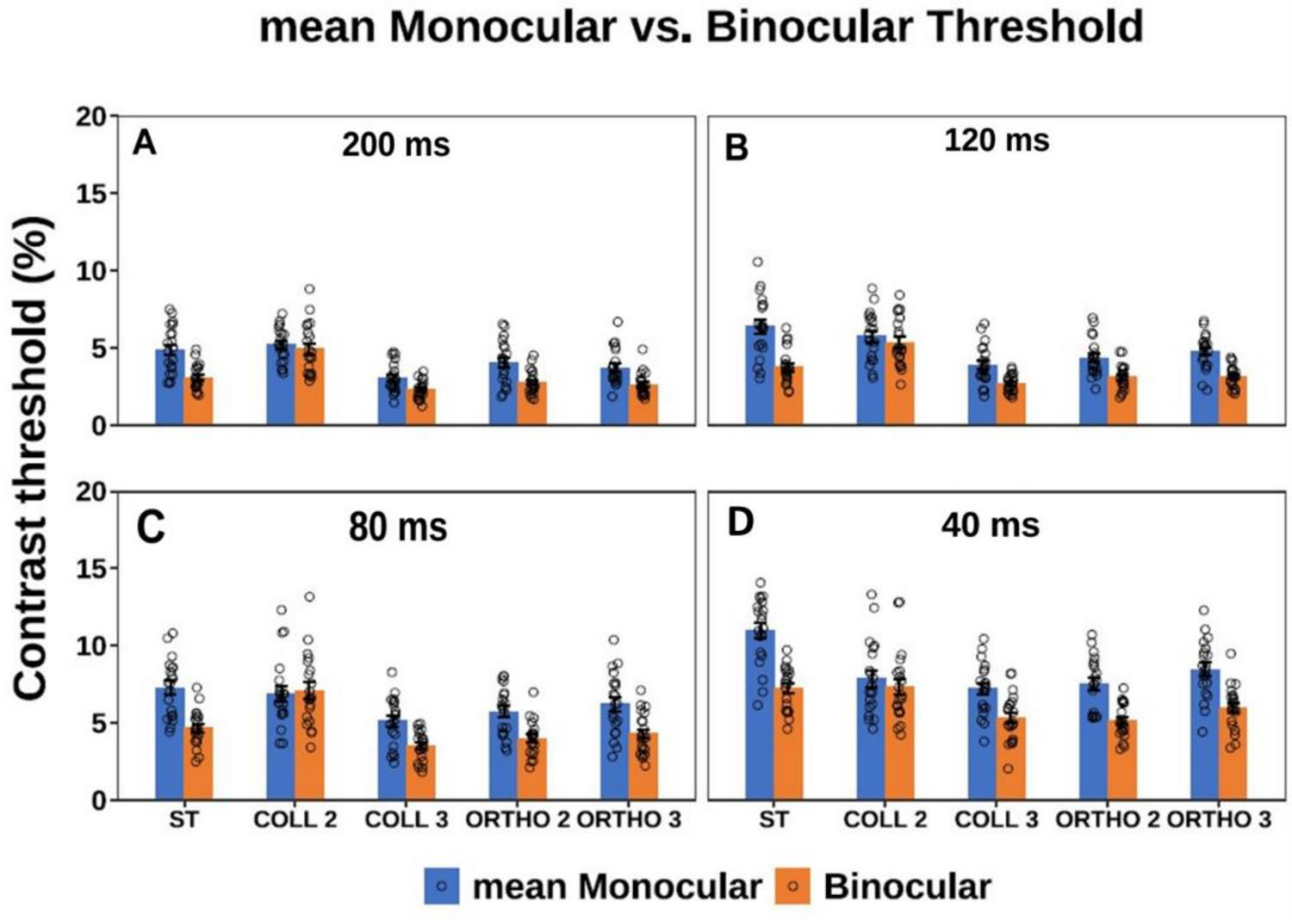
The mean Monocular and Binocular thresholds according to presentation times of 40, 80, 120, and 200 ms under the LM paradigm for 20 different participants averaged from experiment1, control A, control B, and control C experiments. Single Target (ST), Collinear configuration (COLL), Orthogonal configuration (ORTHO) with target–flanker separations of 2 and 3λ for each configuration. (**A**) 200 ms. (**B**) 120 ms. (**C**) 80 ms. (**D**) 40 ms. N = 20, Error bars represent the standard error of the mean (SEM).

In other words, in order to summarize the results of BS across the 4 different experiments (exp.1, control A-C), we averaged all conditions as a mean value of BS for all 4 different presentation times for each stimulus condition at each individual experiment, and then since there is no significant difference between the 4 different experiments for each stimulus condition (see Fig. 11A), we averaged the BS between the 4 different experiments for each stimulus condition (see Fig. 11B). Figure 12 presents the mean monocular and binocular thresholds of presentation times of 40, 80, 120, and 200 ms under the LM paradigm for 20 different participants averaged from the experiment 1, control A, control B, and control C experiments. There are no differences in binocular summation between the different presentation durations.

## Discussion

The aim of our study was to investigate the effect of spatial and temporal domains on binocular interactions and the BS phenomena. We investigated how BS is affected by binocular interactions, by the testing order, and by practice under different space and time conditions using the LM paradigm. We found that BS is not uniform (1.4); it depends on the testing conditions, the presentation time, and the methods used to control the monocular and binocular vision. Our results can be explained by the dynamics of binocular interactions (suppression and/or facilitation): interocular (between eyes) and local (monocular within each eye). The results show that BS is a combination of both types of interactions, suppression and/or facilitation, depending on the target-flanker separations either at 2 or 3 wavelengths (λ).

Importantly, our results pose an intriguing question regarding the optimal BS testing method. The testing order affects the binocular interactions and the BS under the collinear 2λ condition. During each experiment, one variable was tested: either the order of the stimuli's presentation time was displayed from the longer to shorter or vice versa, or mixing the presentation time, or the order of the spatial conditions was displayed (collinear 2λ before 3λ). Thus, the BS under the collinear 2λ condition is not uniform and it depends on the testing order; in other words, the BS is dynamic under the collinear 2λ condition, whereas at a single target, collinear 3λ, and under the orthogonal 2 and 3λ conditions it is uniform.

Several studies found^64,65,95^ that collinear interactions at 3λ affect the collinear interactions at 2λ, leading to reduced lateral suppression. Furthermore, it was reported that practice modifies the range of lateral interactions^96^. The two processes (interactions and practice) lead to dynamic changes in lateral interactions. Since binocular interactions are affected by the monocular interaction^23,89^, BS is also affected by the two processes described above.

Since it has been shown that BS is significantly affected by the spatial and temporal frequency of the stimulus^10^, in our study we chose a constant spatial frequency (8 cpd) to explore the temporal domain on binocular interactions and BS. Here we aimed to investigate how binocular interactions, hence BS, are affected by both collinear and non-collinear interactions under different space and time conditions using the LM paradigm. We used opaque lenses to control the monocular and binocular vision. We hypothesized that BS is not uniform; it depends on the stimuli's presentation time order, practice, and the target-flanker separation (either at 2 or 3λ). In agreement with our hypothesis, we found that the BS of a contrast threshold is absent at close distances (2λ), depending on the presentation time’s order, for the collinear but not for the orthogonal configuration. However, the BS of contrast threshold also exists at more distant flankers (collinear and orthogonal, 3λ).

Note that for the single target condition, 72.5% of the participants reached BS when the BS ratio was ≥ 1.4, whereas 27.5% of the participants reached a BS ratio between 1 and 1.4 (1 ≤ BS < 1.4). However, under the collinear 2λ condition, only 16.25% of the participants reached BS ≥ 1.4, depending on the stimuli's presentation time order (see Fig. 11B), whereas 43.75% of the participants reached a BS ratio between 1 and 1.4 (1 ≤ BS < 1.4). Interestingly, 40% of the participants reached BS < 1 (suppression) probably due to the inter-ocular suppression effect. Importantly, under the collinear 3λ condition, 52.5% of the participants reached BS (the BS ratio was ≥ 1.4), whereas 36.25% of the participants reached a BS ratio between 1 and 1.4 (1 ≤ BS < 1.4). Interestingly, 11.25% of the participants reached BS < 1, probably due to the inter-ocular suppression effect (see Fig. 11B). Our results show that the close distances of the collinear 2λ condition (but not for the orthogonal condition) suppress detection more under binocular than under monocular vision, depending on the stimuli's presentation time order, whereas more distant flankers (collinear 3λ) facilitate both monocular and binocular detection. We found that two eyes are not better than one with crowded targets (the collinear 2λ condition), which is consistent with previous studies^22,23^. In summary, we found that a BS of contrast threshold is absent at close distances (2λ), depending on the stimuli's presentation time order, for the collinear but not for the orthogonal configuration. However, a BS of contrast threshold exists at more distant flankers (collinear and orthogonal, 3λ), which is consistent with previous studies^23,89^.

### BS of collinear facilitation

The BS model^10^ suggests that BS is higher under lower contrast conditions. Thus, since the contrast thresholds under binocular facilitation are lower than those of the single targets, we should expect a higher BS under collinear facilitation. However, our results did not show an advantage of BS under binocular facilitation for all presentation times, showing that the BS values of single targets and under collinear facilitation are approximately equal.

**Figure 13:**
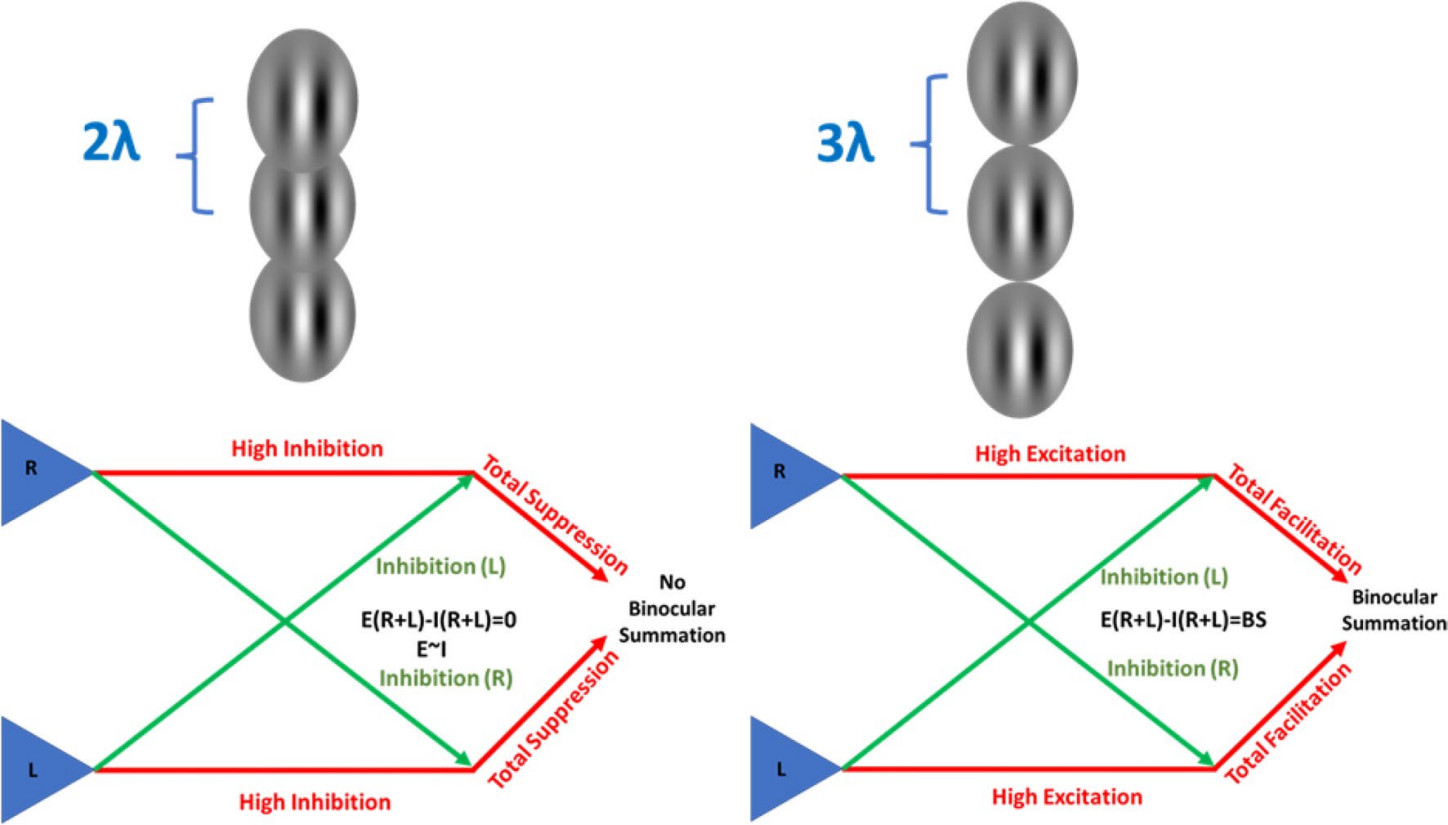
Illustration of a model explaining the monocular and binocular interactions during BS phenomena under the Lateral Masking Paradigm in our study with target–flanker separations of 2 and 3λ.

Our results for monocular and binocular vision under the collinear 2λ condition and the BS of the collinear facilitation effect can be explained by a gain control model^23,25,32,46^, whereby each eye exerts gain control of the signal from the fellow-eye (the interocular inhibition that makes the output a bit "noisy") followed by a summation of them. It is well known that LM can either facilitate or suppress detection, depending on their distance from the target and the global configuration^63,64,68,87,88^. At close distances (collinear 2λ) there are two types of suppression: one type induced by the long-range horizontal connection within V1 (lateral interactions); the other one is induced by the interocular suppression. When the two types of suppression act together, detection is suppressed more under binocular than under monocular viewing. In contrast, more distant flankers (collinear 3λ) facilitate both monocular and binocular detection, due to the collinear facilitation at 3λ. Indeed, interocular suppression eliminates the collinear facilitation effect induced by lateral interactions and then equals the collinear facilitation induced under the monocular and binocular conditions (see Fig. 13).

#### Monocular versus binocular lateral interactions

Our results did not show an advantage in binocular collinear facilitation for all presentation times, suggesting that binocular collinear facilitation is approximately equal to the amount of collinear facilitation of the eye with the better collinear facilitation, which is consistent with previous studies^23,89,91^. We found collinear facilitation at all presentation times at larger distances (collinear 3λ). For closer distances (collinear 2λ), the facilitation depends on the presentation time and the testing order of the stimuli. Our results are consistent with those in the literature^63–65,81^ showing that facilitation exists for collinear configuration at a target-flanker separation of 3λ, but differs at 2λ; thresholds may be elevated (suppression) for shorter target-flanker separations. In summary, the BS phenomenon of collinear facilitation is absent for close or far stimuli (collinear 2 and 3λ), whereas it is intact for isolated stimuli (a single target), which is consistent with previous studies^23,89,91^.

#### Dynamics of binocular interactions and BS at close distances—the collinear 2λ condition

There might be a few possible views that attempt to explain the dynamics of binocular interactions and BS at a close distance under the collinear 2λ condition.

(1) Fast learning within sessions; improvement that can be affected by the number of trials^101,102^ on a time scale of a few minutes when the input provided is of high quality.

(2) Slow learning (day-to-day improvements); performance improves^103^ several hours after, but not during or shortly following the practice session (the latent learning phase). This stage includes the terms “memory consolidation” and “long term memory”^104–111^. We suggest the term “consolidation” for this process, which could begin during the first session, which underlies the improvement in perceptual sensitivity several hours after visual experience ended, and it results in very long-term memory.

In our study we assumed that performance has improved after a normal night’s sleep; some studies^112–114^ found that sleep was critical for perceptual learning. The relationship between fast and slow learning remains unclear; however, they may involve different stages (levels) of visual processing.

(3) Testing order and practice; they may explain the dynamics of binocular interactions and BS, since it was found^64,65,95^ that collinear interactions at 3λ affect the collinear interactions at 2λ, leading to reduced lateral suppression; therefore, it has been reported that practice with collinear configuration increases the facilitation, possibly due to improvement of the synaptic efficacy^2,64,65^. Furthermore, it was reported that practice modifies the range of lateral interactions^96^. The two processes (interactions and practice) lead to dynamics in the range of lateral interactions. The binocular interactions are affected by the monocular interaction^23,89^; therefore, BS is also affected by the two processes described above.

(4) Contrast summation; an alternative explanation for the dynamics of binocular interactions and BS under the collinear 2λ condition is summation of the flanker and the target contrasts by a single large filter (the dipper function). Several studies^115,116^ proposed that the facilitation effect is due to contrast summation of the target and the flankers by a single large filter. Consistent with this view is the suggestion that the visibility of the flanker is a critical parameter, which in cases of low visibility, would impair the contrast facilitation^117^.

Polat^85^ found that the transducer function cannot account for the collinear facilitation effect. A later study^96^ provided additional data that cannot be consistent with the explanation of collinear facilitation as a transducer function; despite the large difference between the flanker contrasts, the magnitude of facilitation remains comparable. Note that in our data, the flanker contrast (60 or 90%) that we used was 6 times above the target’s contrast threshold (10 or 15%), which should shift the facilitation to suppression^118^; however, this effect was sometimes not found (the dynamics of collinear interactions). Therefore, we concluded that it is unlikely that the effect of facilitation is due to a summation of the target and flanker contrast from inside the linear filters. Interestingly, a recent study^94^ found that the effect of canceling the collinear facilitation is consistent with a previous study^85^, which contrasts with the explanation of collinear facilitation as a transducer function. Note also that our data are not consistent with the suggestion of dependency between the flanker visibility and facilitation^117^. Our data show that facilitation is evident once the flanker contrast reached contrast values about twice the target threshold. Moreover, in our recent study^119^, collinear facilitation was found when the flanker contrast was 2, 4, 6, 8, and 10 times above the target’s threshold, which is consistent with facilitation at 3λ of contrast detection relative to the target condition^63,68,85,118,120^. This effect could be found because the facilitatory effects are largely contrast independent when the flanker contrast is more than twice the detection threshold of the target^63,68,85,116,120^.

In conclusion, we suggest that consistent with recent studies^58,62,120,121^, facilitation is evident regardless of the flanker visibility. Thus, it is unlikely that the dynamics of collinear interactions at 2λ is due to contrast summation of the target and flanker contrast within a larger receptive field.

In summary, practice improves visual performance, an effect known as perceptual learning due to the plasticity of the visual system^2,96,112,122,123^. Practice on collinear interactions has been successful in enhancing the range of facilitation in human adults^2,64,65,96,122^. Much of our knowledge about collinear interactions is based on measuring the visual functions taken from experienced participants. Since the effect of collinear interactions on binocular interactions and BS across different spatial and temporal frequencies is not documented, the effect of spatial and temporal frequency on binocular interactions and BS in a natural state is not well known. Therefore, measuring the effect described in this study from naïve participants is very important.

### BS under spatial and temporal conditions

The aim of our study was to investigate the effect of spatial and temporal domains on binocular interactions and BS. It was reported that contrast sensitivity is reduced with increasing spatial frequency^124^. Baker et al.^10^ have shown that BS is significantly affected by the spatial and temporal frequency of the stimulus. They defined a measure of “stimulus speed” that they calculated as the ratio between stimulus temporal (the presentation time) and the spatial frequency. According to this ratio, a slow speed (including high spatial and low temporal frequencies) will lead to a higher BS. Note that in our experiments the stimuli of Gabor patches (GPs) were presented at 4 different presentation times of 40, 80, 120, and 200 ms with a constant spatial frequency (SF) of 8 cycles per degree (cpd) across all the different experiments. Therefore, the speed (the ratio of spatial to temporal frequency) was 3.125, 1.562, 1.041, and 0.625 in (deg/s) at the 4 different presentation times of 40, 80, 120, and 200 ms, respectively. For all the experiments, our results showed, in general, a constant effect of BS for the different presentation times (no significant effect of presentation time was found; see Figs. 3, 4, 5, 6 and 11) at each stimulus condition such as a single target, collinear, and orthogonal configurations at target-flanker separations of 2 and 3λ. However, these results contradict the expectations based on the suggestion that low temporal frequency increases BS^10^. This difference could be explained by the different spatiotemporal properties of the stimuli used in our study. Therefore, here the comparison of stimulus speed may be less relevant for our study. We also noted that we used a local Gabor patch as a stimulus, compared to a sine wave grating stimulus with a higher number of cycles, which might not be smoothed by a Gaussian filter, whereas many studies^10^ used a single E target, with differences in size, times of exposure, and contrast, or horizontal Gabors or grating.

In summary, for all the experiments, our results showed that BS is typical for the different presentation times at each stimulus condition such as a single target, collinear, and orthogonal configurations at target-flanker separations of 2 and 3λ. One reason could be that the two eyes do not fully align (a small phase shift between the eyes) on the vertical Gabor, which creates a difference in the phase shift in each eye during binocular viewing, consequently impairing the BS^25,125,126^.

A recent study^89^ in our lab shows that for the heterophoria group, the BS is typical for the horizontal meridian but not for the vertical meridian, which shows no BS. Indeed, the binocular contrast thresholds of a single target in the vertical meridian were almost equal to those of the monocular thresholds. One reason could be that the two eyes do not fully align (a small phase shift between the eyes) on the vertical Gabor, which creates a difference in the phase shift in each eye during binocular viewing, consequently impairing the BS^25,125,126^.

#### Effect of mixing the conditions (i.e., the presentation times) between the eyes

The results of mixing the conditions between eyes (i.e., switching between eyes each time while covering the other), raises challenging questions regarding the BS mechanisms. Using this method in the pilot resulted in large variability and unstable results. Therefore, in this study we first tested all parameters in one eye before switching to the other. To further explore this intriguing question, we initiated an additional study using dichoptic googles in which the participants were unaware of the eye that perceives the stimuli. Interestingly, the revealed pattern of the results is different. The BS is present for all presentation times under all conditions, also for collinear 2λ. Importantly, when we performed the experiment with the mixed eye condition (simultaneously), BS exists at collinear 2λ. Therefore, we suggest that BS may be affected by additional factors that we have not yet considered. We believe that a discussion of these results in light of our questions regarding BS may result in no simple answer. We are analyzing the results, and a draft of the paper is in preparation.

#### Mechanisms underlying BS and a descriptive model

The impairment of BS with closed collinear flankers shares certain characteristics with several well-documented flanking phenomena that modulate the visibility of stimuli, among them, crowding, masking, and surround suppression^22,121,128–130^. For example, BS is orientation specific and depends on target-flanker separations either at 2 or 3λ. Importantly, in our study we investigated the effect of spatial and temporal domains on binocular interactions and BS under different space and time conditions using the lateral masking paradigm. We found, as illustrated by our Descriptive Model (see Fig. 13), that BS is a combination of monocular lateral interactions (suppression and/or facilitation), depending on whether the target-flanker separation is either at 2 or 3λ, and the interocular suppression. For 2λ the output of lateral interactions is shifted toward suppression, thus combined with the interocular suppression, the net BS is abolished.

Our results for a single target, collinear 3λ, and orthogonal 2 and 3λ conditions are consistent with the gained enhancement in the masking model of Meese and Baker^48^, which was suggested to explain contrast detection facilitation at threshold levels, where uncertainty reduction might have contributed. It is also consistent with the gain control model of collinear facilitation suggested in the recent study of Lev et al.^23^.

Over the years, the computational models of binocular summation and interactions have become quite complex. However, recent models provide simpler explanations for the general effect of collinear 2λ. Meese, Challinor, and Summers^127^ suggested the use of superimposed pattern masks for a very simple interpretation of the loss of binocular summation from masking (when the mask is outside the excitatory range of the detecting mechanism). When masking is absent, the observer benefits from signals in two eyes compared with one, but when the mask contrast is high, and consequently, there is larger masking, there is no binocular advantage. Other models, including the gain control model of Lev et al.^23^, have also successfully captured the abolished BS by nearby flankers.

Many pedestal models operate through gain control. A simple pedestal model operates by combining the target and partial flanker signals from one eye; they operate together to gain control of the other eye. When the contrast of the lateral mask is high or when the superimposed grating (pedestal) abolishes the binocular advantage, there is no binocular advantage^26,133^. As explained above, contrast threshold detection without nearby flankers can be explained by a gain control model^23^ or by masking under the rules for summing contrast within suppressive pathways^127^. Thus, when masking or flankers (that may contribute to suppressive input) are absent, the observer benefits from signals in two eyes compared with one and BS exists. However, adding nearby flankers at a low target contrast (contrast detection) abolishes the BS^23^. A similar effect is expected for high-contrast masking^127^. Thus, regardless of the detailed model’s assumption supported by experimental data, it is now well accepted that BS can be abolished by the context^22,23,127^.

One may suggest that abolishing the BS at 2λ is due to the observer’s task shift from contrast detection to contrast discrimination (or the pedestal effect). At 3λ, a spatial distance that reveals BS, the observer’s task is to detect the low target’s contrast, whereas 2λ is a spatial distance at which the flanker’s and the target’s contrast may be summed within the same receptive field. The study of Lev et al.^23^ tested BS using both contrast detection and contrast discrimination (matching). For both tasks the results were the same. Specifically, for contrast discrimination, they show that even for a single target, without flankers, there is BS for low but not for high contrast. Moreover, the same result was revealed with non-overlapping flankers at 3λ (the periphery is at 4 deg of eccentricity); there is summation for low contrast but not for high contrast. Thus, the effect of BS is determined by the contrast level but not by the task type. They also present a good model that captured these results.

The above studies consider the BS effect as static. Here we found that the order of the stimuli's presentation time affects the binocular interactions and the dynamics of BS under the collinear 2λ condition. In our study, the fact that BS recovers at collinear 2λ, depending on the presentation order, highlights the possibility that lateral interaction of excitation from 3λ. which reduces the suppression (masking effect) is more likely to explain our results. The study of Lev et al.^23^ tested this issue, showing that a gain control theory can possibly explain the absence of BS under the collinear condition; it strongly suggests that the interactions take place before the site of binocular combination. Thus, lateral suppression may cancel or reduce the binocular facilitation. Thus, our suggestion is consistent with the computational models of Meese^127^ and Lev^23^.

## Summary and conclusions

We found that BS is not uniform (1.4); it depends on the testing conditions, the presentation time, and the methods used to control the monocular and binocular vision. This result can be explained by the dynamics of binocular interactions (suppression and/or facilitation): interocular (between eyes) and local (monocular within each eye). Thus, BS is a combination of both suppression and/or facilitation, depending on target-flanker separations either at 2 or 3 wavelengths (λ). Indeed, the testing order affects binocular interactions and BS under the collinear 2λ condition; either the stimuli's presentation time order during the experiment was displayed from the longer to shorter presentation time or vice versa or mixed by the presentation time; the order of the stimuli’s conditions was displayed (collinear 2λ before 3λ). Under the collinear 2λ condition the BS is not uniform and it depends on the testing order; in other words, the BS is dynamic under the collinear 2λ condition, whereas at a single target, collinear 3λ, and under the orthogonal 2 and 3λ conditions the BS is uniform.

### Supplementary Information

Supplementary Information.

## Data Availability

The datasets used and/or analyzed during the current study are available from the corresponding author on reasonable request.
