# Supplementary material for: Space, time, and dynamics of binocular interactions
Source: Sci Rep. 2023 Dec 5;13:21449. doi: 10.1038/s41598-023-48380-2 (PMC10698014; doi:10.1038/s41598-023-48380-2)
Supplement: Supplementary file 1 — Supplementary Information. [file 41598_2023_48380_MOESM1_ESM.docx]

# **Supplementary Material**

**Table 1:** Clinical optometric information of normal subjects. XP Exophoria, EP Esophoria.

***Table 1: Pre-clinical optometric tests****.* ***(A).*** *Monocular and binocular visual acuity were measured by ETDRS (log-MAR chart).* ***(B)****. Refractive corrections.* ***(C).*** *Stereoscopic vision exam.* ***(D)****. The dominant eye was evaluated by the forcing monocular vision technique.* ***(E).*** *Binocular* deviations *were determined with the ‘cover test’ method; occlusion of the good eye revealed the type and direction of Tropia.* The angle of phoria was measured by the alternating cover test (ACT).

# **Results**

## **Control Experiment A: Shorter to longer presentation time**

We performed a three-way ANOVA to determine the effect of presentation time, stimulus condition, and group {mean monocular or binocular viewing} on the contrast detection threshold.

**
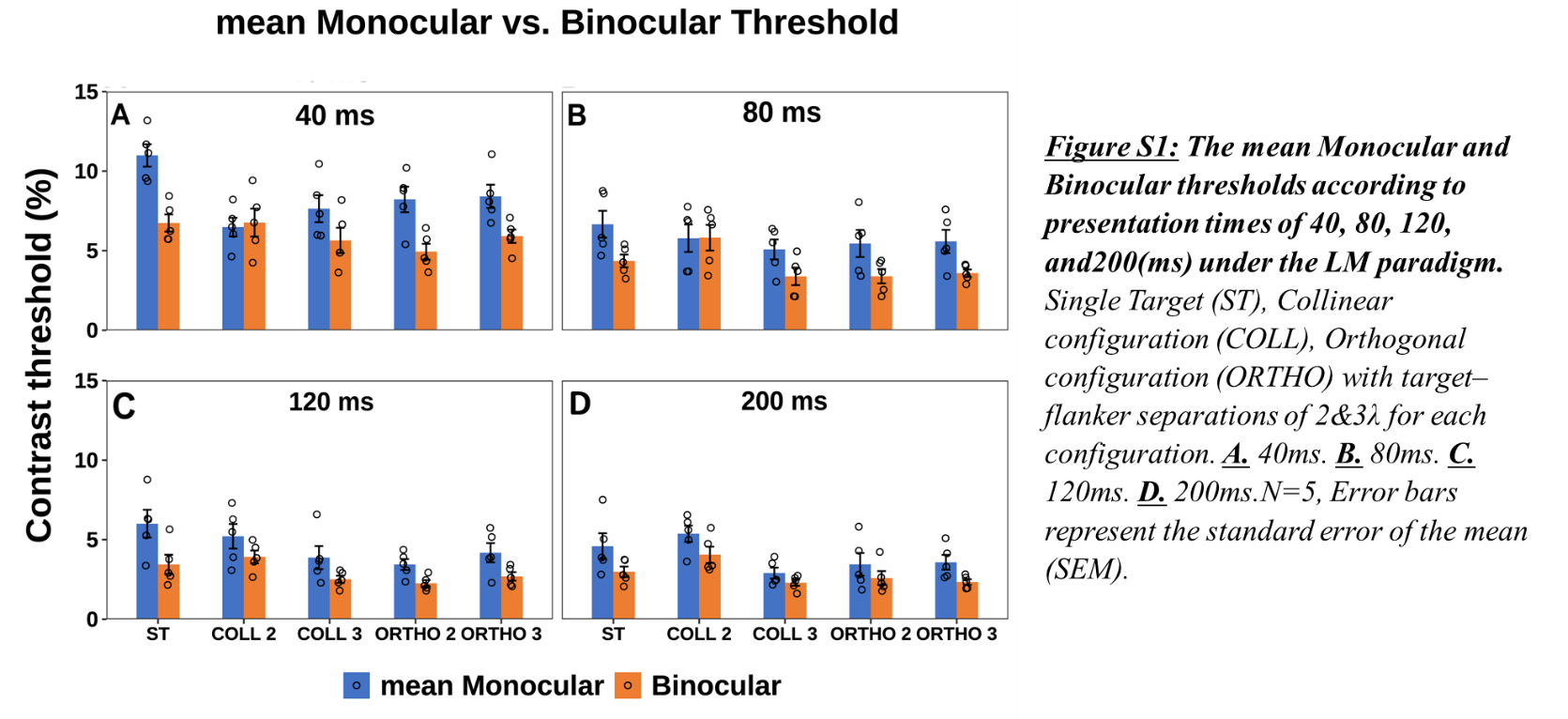
**There was a significant effect of the presentation time (F (3,156) =156.5, p=0), stimulus condition (F (4,156) =22.2, p=0), and group {mean monocular or binocular viewing} (F (1,156) =150.27, p=0) on the contrast threshold. There was a significant interaction between the effect of the presentation time and the stimulus condition (F (12,156) =2.93, p=0.001). Also, there was a significant interaction between the effect of the stimulus condition and group (F (4,156) =6.34, p=0.0001), and there was a significant interaction between the effect of the presentation time and group (F (3,156) =3.48, p=0.02). Specifically, there was a significant difference between the mean monocular and binocular contrast threshold under the single target condition at presentation times of 200, 120, 80, and 40ms (p=0.03, p=0.0003, p=0.001, p=0 by Tukey’s post-hoc analysis, respectively), which could be explained by the BS effect.

***Figure S1: The mean Monocular and Binocular thresholds according to presentation times of 40, 80, 120, and200(ms) under the LM paradigm.*** *Single Target (ST), Collinear configuration (COLL), Orthogonal configuration (ORTHO) with target–flanker separations of 2&3λ for each configuration.* ***A.*** *40ms.* ***B.*** *80ms.* ***C.*** *120ms.* ***D.*** *200ms.N=5, Error bars represent the standard error of the mean (SEM).*

## **Control Experiment B: 2λ before 3λ (longer to shorter presentation times)**

We performed a three-way ANOVA to determine the effect of presentation time, stimulus condition, and group {mean monocular or binocular viewing} on the contrast detection threshold.

There was a significant effect of presentation time (F (3,156) =77.72, p=0), stimulus condition (F (4,156) =49.4, p=0), and group {mean monocular or binocular viewing} (F (1,156) =69.87, p=0) on the contrast threshold. There was a significant interaction between the effect of the presentation time and the stimulus condition (F (12,156) =2.3, p=0.009). Also, there was a significant interaction between the effect of the stimulus condition and group (F (4,156) =9.35, p=0). Specifically, there was a significant difference between the mean monocular and binocular contrast threshold under the single target condition at presentation times of 120, 80, and 40ms (p=0.0006, p=0.002, p=0 by Tukey’s post-hoc analysis, respectively), which could be explained by the BS
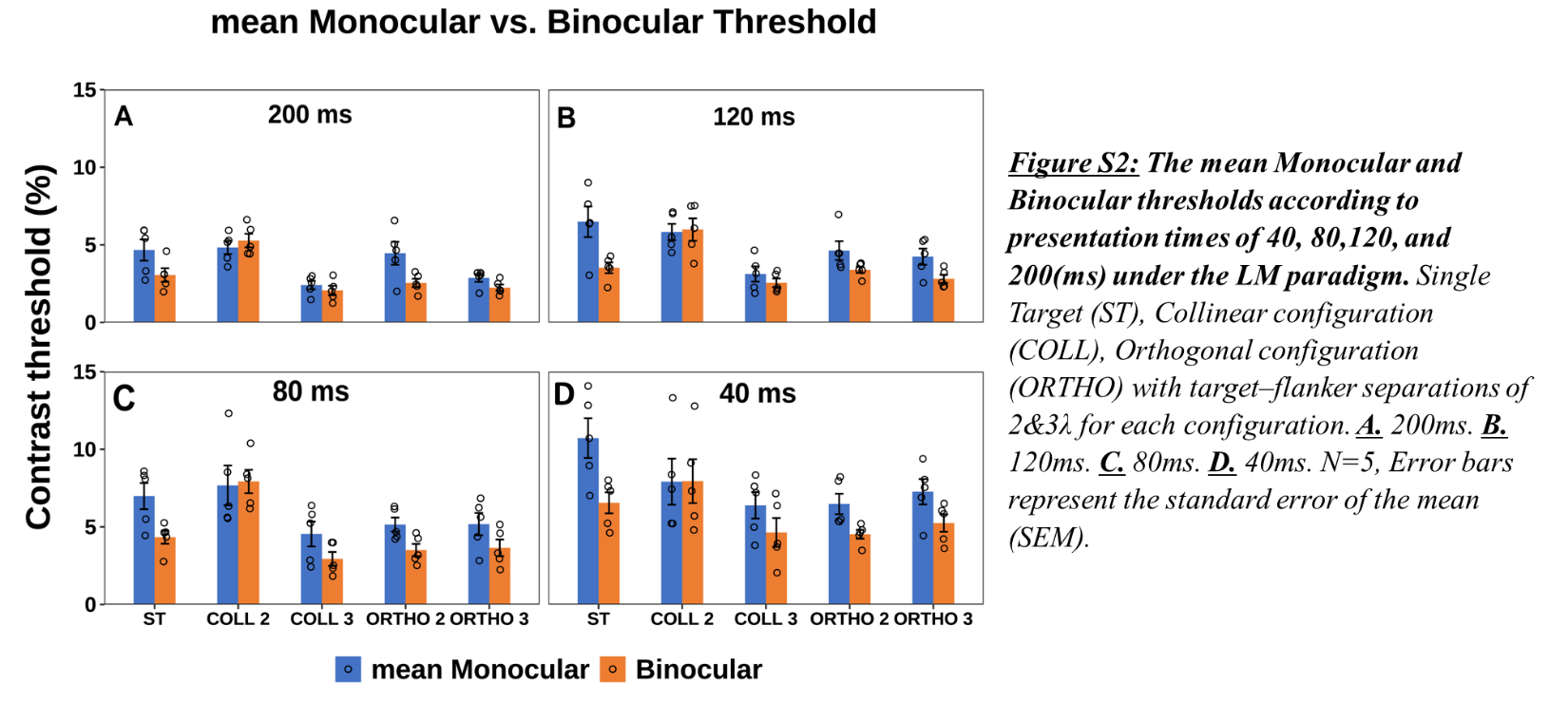
effect.

***Figure S2: The mean Monocular and Binocular thresholds according to presentation times of 40, 80,120, and 200(ms) under the LM paradigm.*** *Single Target (ST), Collinear configuration (COLL), Orthogonal configuration (ORTHO) with target–flanker separations of 2&3λ for each configuration.* ***A.*** *200ms.* ***B.*** *120ms.* ***C.*** *80ms.* ***D.*** *40ms. N=5, Error bars represent the standard error of the mean (SEM).*

## **Control Experiment C: Mixed procedure by the presentation time**

We performed a three-way ANOVA to determine the effect of presentation time, stimulus condition, and group {mean monocular or binocular viewing} on the contrast detection threshold.

There was a significant effect of presentation time (F (3,156) =83.8, p=0), stimulus condition (F (4,156) =20.95, p=0) and group {mean monocular or binocular viewing} (F (1,156) =60.84, p=0) on the contrast threshold. There was a significant interaction between the effect of the presentation time and the stimulus condition (F (12,156) =2.34, p=0.0087). Also, there was a significant interaction between the effect of the stimulus condition and group (F (4,156) =4.94, p=0.0009). Specifically, there was a significant difference between the mean monocular and binocular contrast threshold under the single target condition for presentation times of 40 and 80ms (p=0.003, p=0.005 by Tukey’s post-hoc analysis, respectively), which could be explained by the
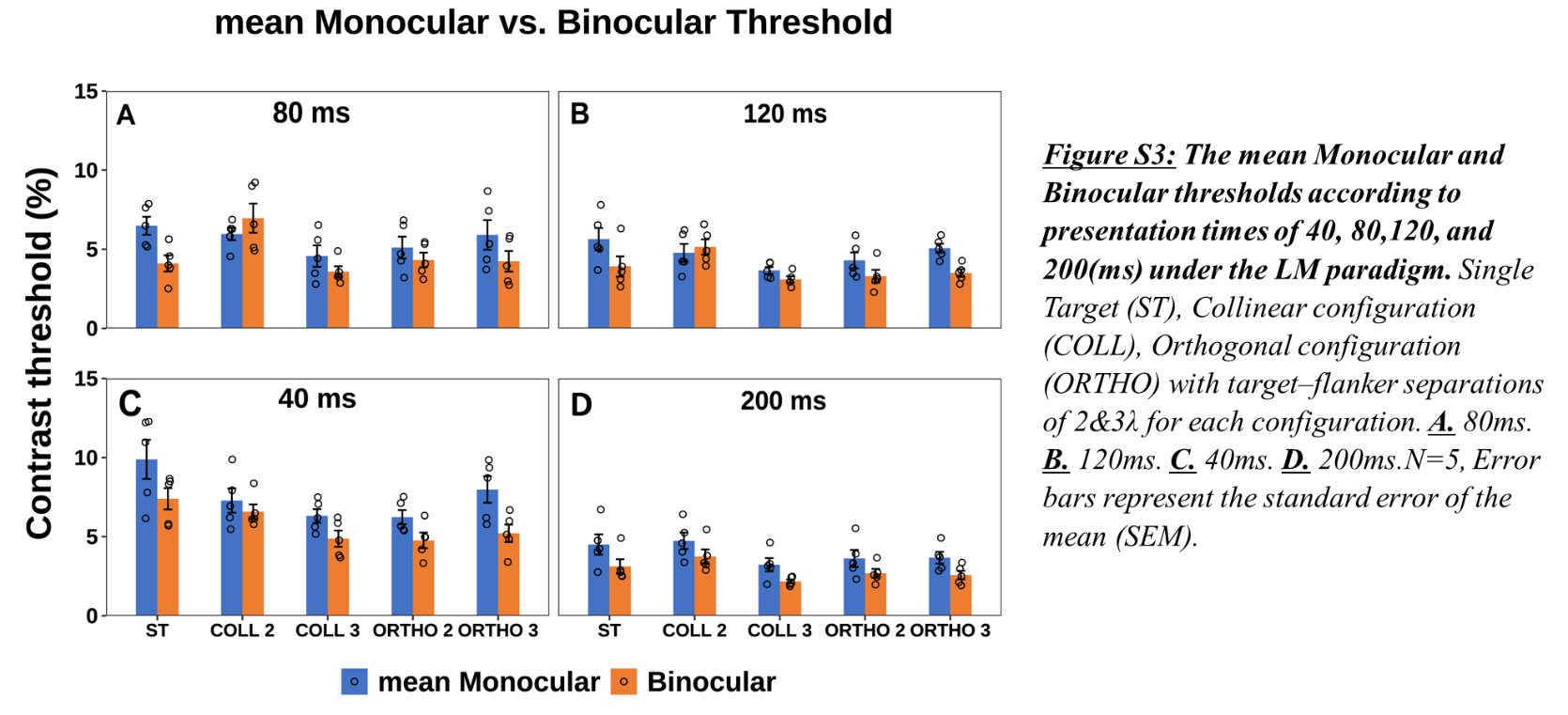
BS effect.

***Figure S3: The mean Monocular and Binocular thresholds according to presentation times of 40, 80,120, and 200(ms) under the LM paradigm.*** *Single Target (ST), Collinear configuration (COLL), Orthogonal configuration (ORTHO) with target–flanker separations of 2&3λ for each configuration.* ***A.*** *80ms.* ***B.*** *120ms.* ***C.*** *40ms.* ***D.*** *200ms.N=5, Error bars represent the standard error of the mean (SEM).*

# **Contrast threshold as a function of presentation time**

**
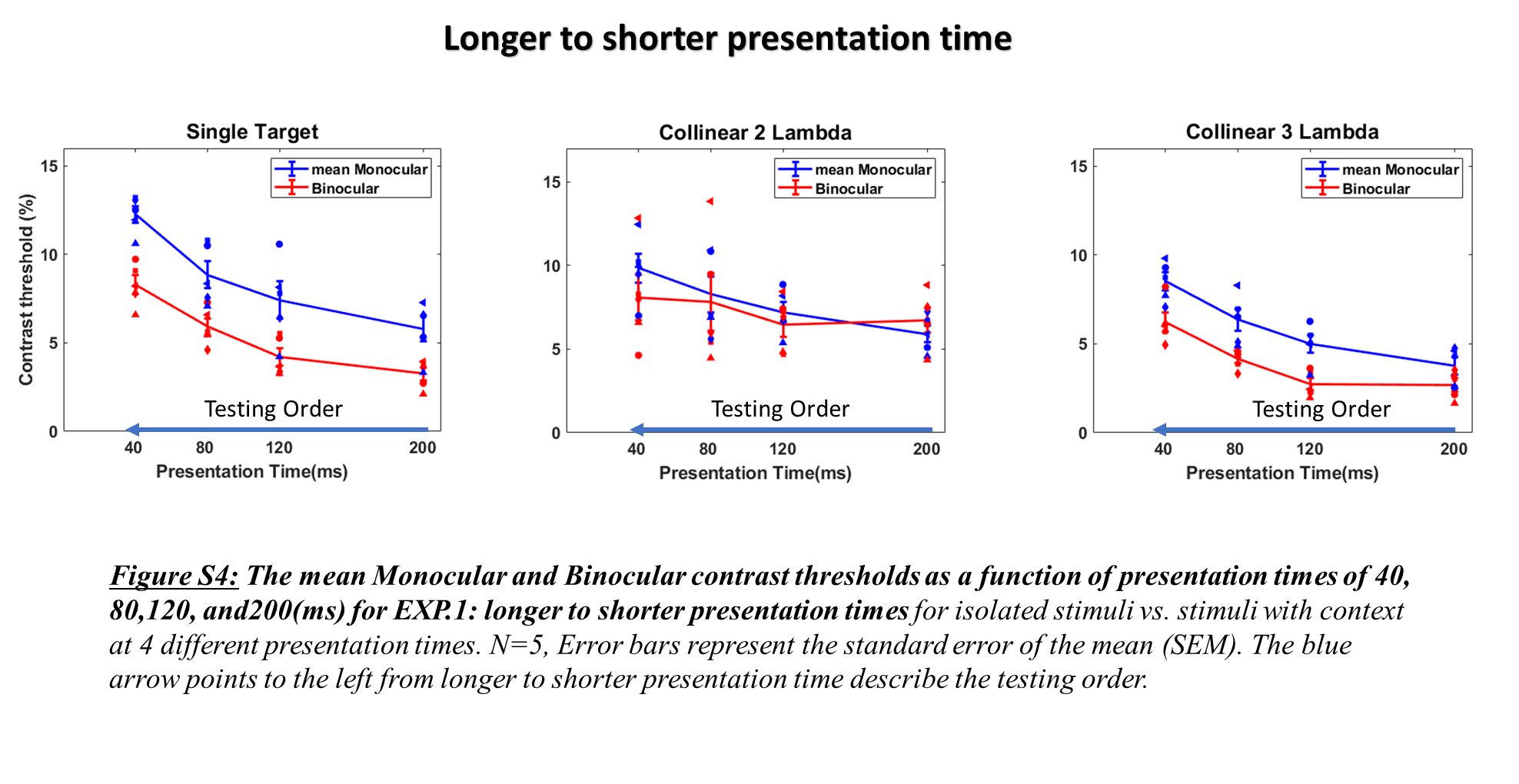
**

***Figure S4: The mean Monocular and Binocular contrast thresholds as a function of presentation times of 40, 80,120, and200(ms) for EXP.1: longer to shorter presentation times*** *for isolated stimuli vs. stimuli with context at 4 different presentation times. N=5, Error bars represent the standard error of the mean (SEM). The blue arrow points to the left from longer to shorter presentation time describe the testing order.*

**
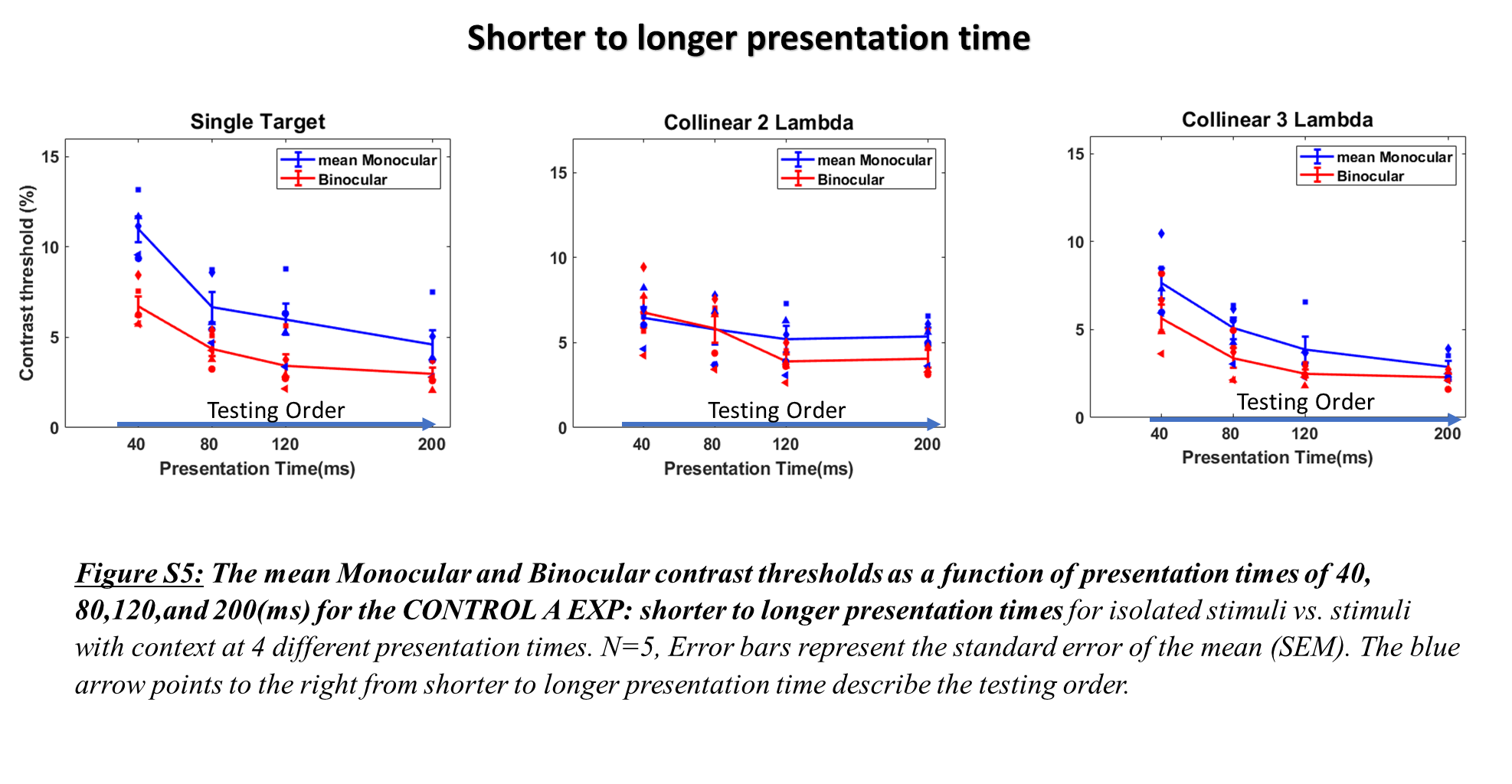

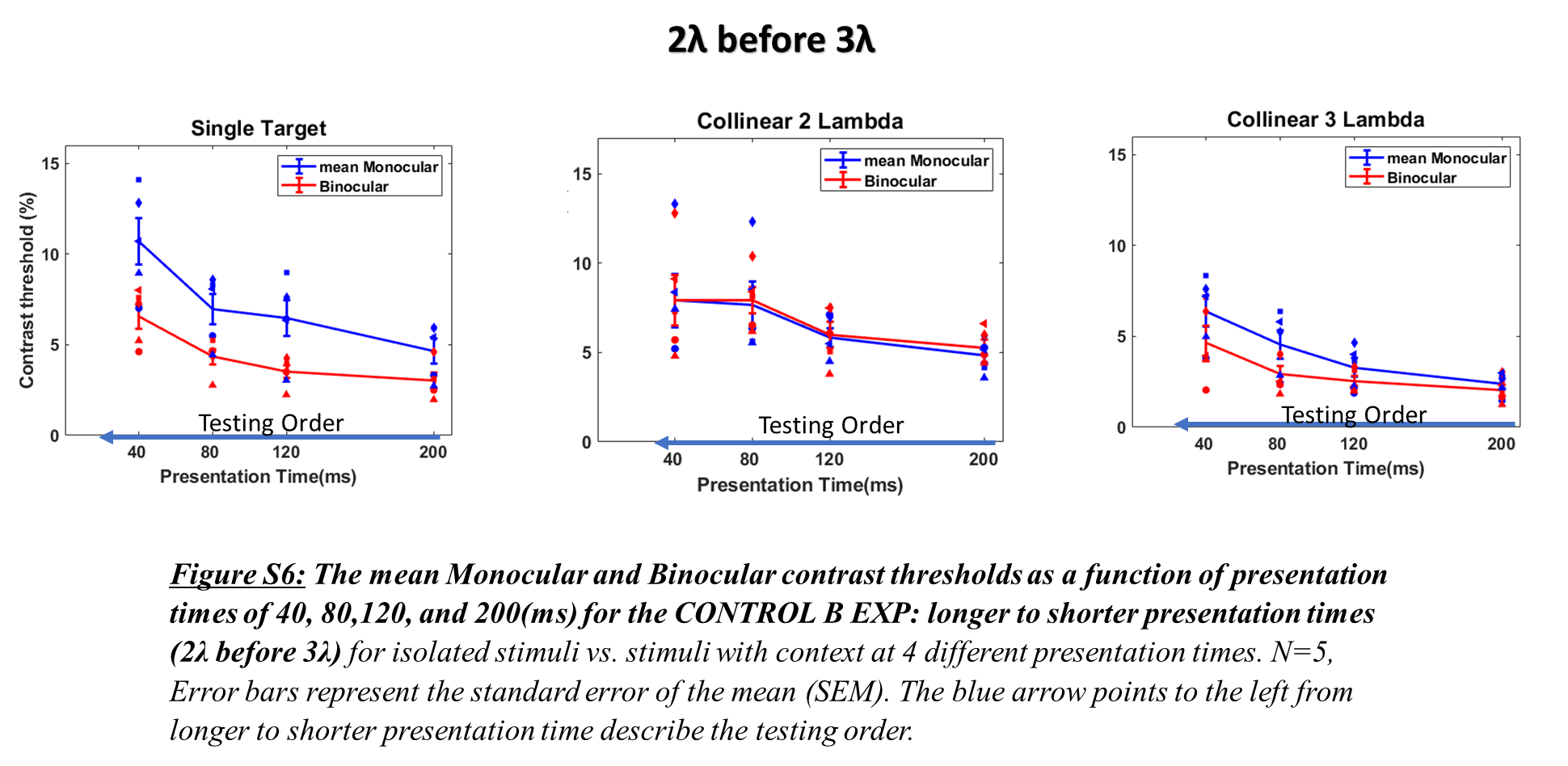
**

***Figure S5: The mean Monocular and Binocular contrast thresholds as a function of presentation times of 40, 80,120,and 200(ms) for the CONTROL A EXP: shorter to longer presentation times*** *for isolated stimuli vs. stimuli with context at 4 different presentation times. N=5, Error bars represent the standard error of the mean (SEM). The blue arrow points to the right from shorter to longer presentation time describe the testing order.*

***Figure S6: The mean Monocular and Binocular contrast thresholds as a function of presentation times of 40, 80,120, and 200(ms) for the CONTROL B EXP: longer to shorter presentation times (2λ before 3λ)*** *for isolated stimuli vs. stimuli with context at 4 different presentation times. N=5, Error bars represent the standard error of the mean (SEM). The blue arrow points to the left from longer to shorter presentation time describe the testing order.*

**
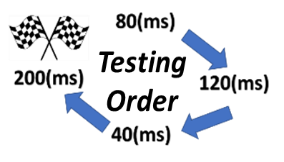

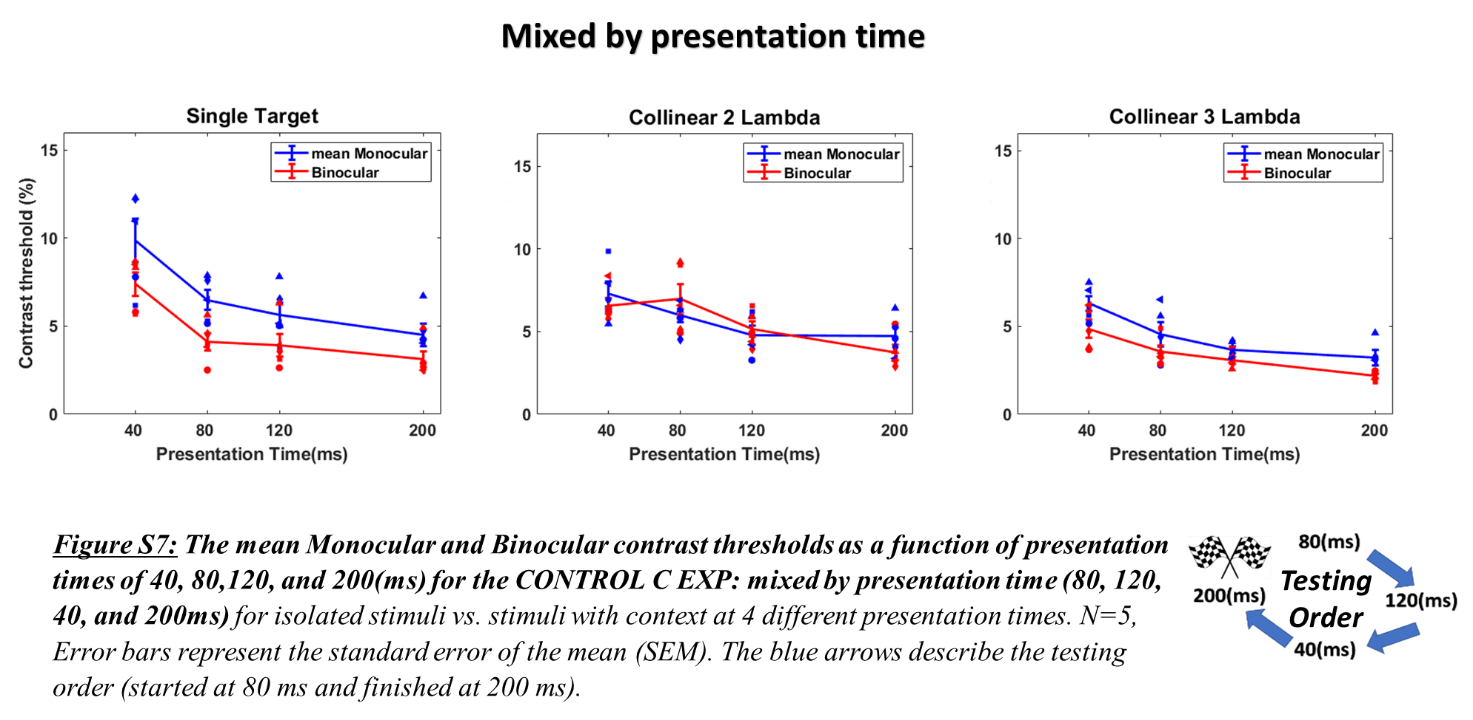
**

***Figure S7: The mean Monocular and Binocular contrast thresholds as a function of presentation times of 40, 80,120, and 200(ms) for the CONTROL C EXP: mixed by presentation time (80, 120, 40, and 200ms)*** *for isolated stimuli vs. stimuli with context at 4 different presentation times. N=5, Error bars represent the standard error of the mean (SEM). The blue arrows describe the testing order (started at 80 ms and finished at 200 ms).*

# **Statistical information**

**Table 2:** Statistical comparison between the **right & left eye for contrast thresholds** under each condition throughout each presentation time during the different experiments. The evaluation was done using post-hoc analysis of 3-way ANOVA. Single Target (ST), Collinear configuration (COLL), and Orthogonal configuration (ORTHO) with target–flanker separations of 2&3λ for each configuration.

**Table 3:** Statistical comparison between the **mean Monocular & Binocular viewing for contrast thresholds** under each condition throughout each presentation time during the different experiments. The evaluation was done using post-hoc analysis of 3-way ANOVA. Single Target (ST), Collinear configuration (COLL), and Orthogonal configuration (ORTHO) with target–flanker separations of 2&3λ for each configuration.

**Table 4:** Statistical comparison between the **BS ratio of the different conditions** throughout each presentation time during the different experiments. The evaluation was done using post-hoc analysis of 2-way ANOVA. In each row, the two parameters represent the two conditions with which the statistical analysis is compared. Single Target (ST), Collinear configuration (COLL), and Orthogonal configuration (ORTHO) with target–flanker separations of 2&3λ for each configuration.

**Table 5:** Statistical comparison between the **mean Monocular & Binocular collinear facilitation (threshold elevation)** at target-flanker separations of 2&3λ throughout each presentation time during the different experiments. The evaluation was done using a post-hoc analysis of 2-way ANOVA. The statistical analysis is compared between the mean monocular and binocular for each condition, which is presented in each row for Collinear configuration (COLL) at target–flanker separations of 2&3λ.

**Table 6:** Statistical comparison between the **Binocular collinear facilitation (threshold elevation)** at target-flanker separations of 2&3λ throughout each presentation time during the different experiments. The evaluation was done using a post-hoc analysis of 2-way ANOVA. The statistical analysis compared the binocular collinear facilitation (the threshold elevation) for collinear configuration (COLL) at target–flanker separations of 2&3λ, which is presented in each row.
